# Caregiver perceptions of healthcare barriers across traditional and digital contexts: a mixed-methods analysis

**DOI:** 10.1038/s41746-025-02131-x

**Published:** 2025-12-12

**Authors:** Jacqueline B. Duong, Sierra N. Walters, Kayla E. Carta, Grace A. Jumonville, Alyssa S. Carrasco, Natalia Simo Fiallo, Daniela N. Romero, Ishita P. Khurd, Matthew W. Ahle, Jonathan S. Comer, Stacy L. Frazier, Theodora Chaspari, Shrikanth Narayanan, Adela C. Timmons

**Affiliations:** 1 University of Texas at Austin, Austin, TX USA; 2 Florida International University, Miami, FL USA; 3 Colliga Apps Corporation, Austin, TX USA; 4 University of Colorado, Boulder, CO USA; 5 University of Southern California, Los Angeles, CA USA

**Keywords:** Health care, Health humanities, Science, technology and society

## Abstract

As digital health expanded during COVID-19, understanding how caregivers perceive differences between traditional and digital care became critical. In a purposive sample of female caregivers of school-age children with elevated mental health symptoms (*n* = 47), we used a convergent mixed-methods design to compare reported experiences across modalities. Thematic analysis revealed that participants described different barrier patterns: financial concerns were reported in both contexts, but with different characteristics (insurance/copays vs. device/subscription costs). Participants reported distinct digital challenges, relational connection loss, digital literacy gaps, and privacy concerns, while facilitators also differed. Traditional care narratives emphasized interpersonal support, while digital care highlighted accessibility and personalization. Social identity intersections were referenced more frequently in traditional care narratives, though demographic correlations suggested continued relevance in digital contexts. These pandemic-era findings from female caregivers generate hypotheses for designing inclusive digital health approaches and highlight areas for longitudinal research tracking actual transitions between care modalities.

## Introduction

Despite extensive research and policy initiatives, health disparities remain a persistent challenge in the United States^1,2^. These disparities are rooted in unequal access to and utilization of healthcare services, disproportionately impacting marginalized communities^3^. The rapid growth of digital health technologies (DHTs), including telehealth, mobile health apps, and remote monitoring, has the potential to bridge these gaps by increasing healthcare access, enhancing patient engagement, and enabling personalized care^4–6^. However, the extent to which DHTs mitigate or reproduce existing barriers, particularly for structurally marginalized groups, remains unclear^7^. This mixed-methods study examines how barriers and facilitators compare across traditional and digital healthcare contexts and how these dynamics intersect with social identities to inform equitable digital health design and implementation.

Health disparities, as defined by the CDC (2024)^8^ and Braveman (2006)^9^, are systematic differences in health outcomes that systematically disadvantage marginalized groups and stem from modifiable social, economic, and structural conditions. These disparities encompass both differences in health outcomes (morbidity, mortality, quality of life) and differences in the social determinants that shape these outcomes^9,10^. This definition distinguishes them from health differences that cannot be addressed through structural interventions. Importantly, this focus on modifiability emphasizes theoretical amenability to structural change, regardless of whether specific interventions are currently implemented.

To understand how these disparities manifest across care modalities, we draw on Schneider-Kamp’s (2021)^11^ conceptualization of *health capital*, which adapts Bourdieu’s field-dependent view of capital to the social field of health, treating health as the aggregate of economic, social, cultural, and symbolic resources that shape individuals’ positions and enable or constrain health practices. From this perspective, social determinants influence health through multiple, context-dependent pathways, which may operate differently across traditional and digital care settings. For clarity, the examples we list below—unequal access to high-quality care^12,13^; community resources and environmental conditions^14,15^; provider discrimination^16,17^; and language or cultural barriers that hinder patient–provider communication^18^—are illustrative, not exhaustive. This perspective emphasizes that disparities arise from layered exposures to socioeconomic disadvantage, systemic discrimination, and unequal access to resources, with this capital-based understanding being particularly relevant when comparing care modalities that may require different combinations of economic, social, cultural, and symbolic resources.

The COVID-19 pandemic both illuminated and exacerbated these existing inequities, with marginalized communities experiencing disproportionately higher infection and mortality rates alongside reduced access to essential care^19,20^. Simultaneously, the pandemic catalyzed unprecedented DHT adoption; telehealth usage increased seventy-eightfold from February to April 2020, peaking at 32% of office visits^21^. However, this expansion revealed significant disparities: Hispanic patients demonstrated 41% lower telehealth use compared to non-Hispanic White patients^22^ and, when accessed, White patients used video connections for 61% of encounters versus only 50% for Hispanic/Latino and Black patients^23^. Rural populations were 42% less likely to use telemedicine despite a greater need due to provider shortages^23^. These patterns suggest that rapid digital health adoption may have inadvertently widened existing healthcare disparities rather than reducing them.

DHTs show considerable promise for reducing health disparities by addressing traditional barriers to care. These tools can reduce travel and scheduling constraints, provide 24/7 availability, and support culturally tailored interventions^24,25^. At the same time, the *digital divide*—defined as systematic differences in resources and capabilities required to access and effectively use digital technologies—poses significant barriers, especially for marginalized and underrepresented groups^26,27^. This divide manifests in multiple dimensions, including physical access to technology, digital literacy, and meaningful use of digital resources for health improvement. For example, while smartphone access is relatively consistent across racial groups, Black and Hispanic adults are less likely than White adults to own laptops or have home broadband access^28,29^. These gaps limit participation in telehealth and other digital services, particularly in low-resource households and rural areas^30,31^. In some cases, digital tools may offer greater convenience for those already accessing care, rather than expanding access to new or marginalized users^32^.

Understanding what facilitates access to healthcare is as important as identifying barriers, especially as healthcare delivery increasingly incorporates digital tools. In traditional healthcare settings, facilitators such as trusted providers, family support networks, and culturally grounded services have been shown to ease access and promote engagement, particularly for communities historically marginalized in the healthcare system^33,34^. In digital contexts, emerging evidence highlights new facilitators, including culturally tailored content, digital literacy support, and user-centered design^27,35,36^. Community-based digital interventions and peer networks have also been found to promote adoption and engagement among underserved populations^35,37^. DHTs may reduce traditional access barriers through increased flexibility, asynchronous communication, and customizable interfaces that accommodate a range of linguistic, cognitive, and physical needs^24,38^. Additionally, digital platforms may reduce barriers related to provider mistrust or facility-related stigma for some populations, with randomized trials showing improved treatment engagement among minoritized families receiving telehealth compared to office-based car^39^. This advantage may be particularly pronounced for families with young children, where telehealth enables treatment in natural home settings during a critical developmental period when children are transitioning to formal schooling^39^. These observations motivate a comparative focus on how facilitation works across traditional and digital settings, rather than assuming direct transfer.

It is also important to consider how barriers and facilitators intersect with social identities. According to intersectionality theory^40,41^, people’s experiences of discrimination are shaped by the combination of their social identities, like race, gender, income, and disability. In healthcare contexts, barriers and facilitators can therefore manifest differently across populations^42,43^. Illustrative challenges include limited funds for devices or data plans, lower digital comprehension among older adults, and language barriers that hinder navigation of digital interfaces. These considerations may be especially compounded for caregivers managing both their own healthcare needs alongside the needs of school-age children. Prior work shows that DHTs can introduce new barriers (e.g., digital access and privacy) and reconfigure existing ones (e.g., costs shifting to devices and data). What remains insufficiently understood is whether and why specific intersections of identity (e.g., race with income or language with caregiving role) are associated with distinct patterns of vulnerability or advantage in digital compared with traditional care, and which combinations are linked to loss of access versus the appearance of new facilitators.

To address this gap, we analyze within-interview comparative accounts and describe reported differences across modalities using a typology: persistence, reconfiguration, and emergence. We also examine how these reported differences vary across intersecting identities and the contextual features frequently cited alongside them in digital settings (e.g., device and broadband availability, private space for care, translation/interpretation supports, digital and eHealth literacy, caregiving responsibilities).

Using a convergent mixed-methods design, we treat the qualitative analysis as primary evidence and use quantitative measures of health literacy, eHealth literacy, technology use, privacy concerns, and identity as descriptive, hypothesis-generating context. Guided by this approach, we ask: (1) Which barriers and facilitators are reported to persist, reconfigure, or emerge when participants compare traditional with digital care; (2) Do the prevalence and forms of these reported differences vary across intersecting identities; and (3) How *does the salience of social identity in healthcare narratives relate to reported barriers and facilitators across traditional and digital contexts?*

## Results

### Demographic information

The final sample comprised 47 female caregivers with diverse linguistic backgrounds: 18 monolingual English speakers (38.3%), 5 monolingual Spanish speakers (10.6%), 19 English-Spanish bilingual speakers (40.4%), 4 speakers of English or Spanish plus other languages (8.5%), and 1 multilingual speaker (2.1%). The sample was racially/ethnically diverse: Hispanic/Latino/a (36.2%), Black/African American (29.8%), Non-Hispanic White/Caucasian (14.9%), Multi-race (10.6%), Other (6.4%), and American Indian/Alaska Native (2.1%). Most participants were employed full-time (57.4%) with low to moderate income levels (80.85%). See Table 1 for more detailed demographic information.

**Table 1:** Sample demographics

Caregiver demographic characteristics	*M* (SD) or *N* *(%)*	Range
Age (year)	41.49 (6.56)	26–56
Female	47 (100%)	
Sexual Orientation		
Straight	46 (97.9%)	
Gay, Lesbian, Bisexual	1 (2.1%)	
Ethnicity		
Hispanic or Latino/a	17 (36.2%)	
Non-Hispanic or Non-Latino/a	30 (63.8%)	
Race		
American Indian/Alaska Native	1 (2.1%)	
Asian	0 (0.0%)	
Black/African American	14 (29.8%)	
Hispanic White	17 (36.2%)	
Multi-race	5 (10.6%)	
Non-Hispanic White/Caucasian	7 (14.9%)	
Other	3 (6.4%)	
Born in the US	34 (72.3%)	
Education level		
Graduate degree	14 (29.8%)	
Bachelor’s degree	14 (29.8%)	
Some college but no degree	7 (14.9%)	
High school degree or equivalent (e.g. GED)	4 (8.5%)	
Associates degree	7 (14.9%)	
Less than high school degree	1(2.1%)	
Employment Status		
Full-time	27 (57.4%)	
Part-time	5 (10.6%)	
Student	2 (4.3%)	
Homemaker	8 (17.0%)	
Currently unemployed	3 (6.4%)	
Retired	2 (4.3%)	
Annual household income before taxes		
$$\le \,$$$50,000 (Low)	24 (52.2%)	
$50,001–99,999 (Middle)	13 (28.3%)	
$$\ge$$ $100,000 (High)	9 (19.6%)	
Not reported	1 (2.12%)	
Preferred language		
English	40 (85.1%)	
Spanish	7 (14.9%)	

### Qualitative analysis

Thematic analysis revealed 18 distinct themes organized across four domains: Traditional Healthcare Barriers (five themes), Digital Health Technology Barriers (four themes), Traditional Healthcare Facilitators (four themes), and Digital Healthcare Facilitators (five themes). Table 2 presents an overview of the identified themes, along with their corresponding frequency counts.

**Table 2:** Theme overview

Domain	Theme	Definition	Exemplar quote	N/47 (%)
Traditional healthcare barriers	1. Cultural Belief Barriers	Cultural norms and family dynamics limit open discussion and help-seeking.	“Because my parents were from South Carolina, you didn’t talk; you kept everything to yourself… I’ve suppressed a lot of stuff [P16].”	44/47 (94%)
	2. Financial Access Constraints	Costs and insurance gaps force trade-offs and delay or deter care.	“I should really go to a therapist, but they’re so expensive… I could put that money toward different things [P28].”	42/47 (89%)
	3. Structural System Limitations	Scheduling, availability, and bureaucracy hinder timely, continuous care.	“It’s very frustrating to find a PCP… I waited a month and a half… making appointments in a timely manner [P36].”	37/47 (79%)
	4. Provider Bias and Discrimination	Perceived bias or stereotyping reduces trust and care quality.	“When it comes to Black women, [providers] don’t go as far to help them… my son cries and they say ‘behavior problem’ [P42].”	35/47 (74%)
	5. Pandemic-Related Disruptions	COVID-19 stressors and closures reduced availability and support.	“My mental health started to affect my body… I wasn’t speaking to my counselor; no time or childcare during the pandemic [P34].”	28/47 (60%)
DHT barriers	1. Relational Connection Loss	Digital visits weaken rapport, communication, and therapeutic alliance.	“I prefer mental health in person… I could deal with it, but I prefer the human connection [P18].”	34/47 (72%)
	2. Digital Literacy and Access Gaps	Devices, internet, and skills barriers limit independent digital use.	“Getting my mom on Zoom; I have to walk her through every step; for people not tech savvy, portals are a barrier [P28].”	21/47 (45%)
	3. Data Security Concerns	Privacy and hacking worries reduce willingness to use tools.	“The concern would be hackers—I’m sure they’ll find a way around it [P37].”	17/47 (36%)
	4. Value-Cost Assessment	Users weigh subscription costs against perceived benefits and coverage.	“To get the most from the app, you have to pay; some are expensive; I’d use them if insurance covered it [P3].”	13/47 (28%)
**Domain**	**Theme**	**Definition**	**Exemplar quote**	**N/47 (%)**
Traditional healthcare facilitators	1. Supportive Relationship Networks	Family and friends provide emotional support and navigation help.	“I have a good network, my sister’s a social worker telling me, ‘Hey, go visit this doctor’ [P26].”	44/47 (94%)
	2. Healthcare Knowledge Brokers	Trusted people with medical know-how guide decisions and access.	“My aunt is a psychiatrist… it’s always been part of my life; if you need help, you ask for it [P10].”	36/47 (77%)
	3. Cultural Resource Integration	Faith and cultural communities provide belonging and practical support.	“People at my church help my family and give my children love… I have a village willing to help; I just have to ask [P33].”	26/47 (55%)
	4. Non-Clinical Digital Resources	Social media and forums help locate care and share experiences.	“I’m buying insulin from people online; there’s a big Facebook network for this right now [P9].”	11/47 (23%)
DHT facilitators	1. Accessibility and Flexibility	Telehealth and apps reduce time, transport, and scheduling barriers.	“I can sit in my living room at 7:15, do my kids’ doctor appointment… it’s made my life so much easier [P29].”	45/47 (96%)
	2. Personalized Health Management	Tracking and tailored content support individual goals continuously.	“Mindfulness, the Breath app on Apple Watch, treadmill or outside walks—any exercise, you can link it [P2].”	27/47 (57%)
	3. Engagement Mechanisms	Goals, feedback, and competition sustain motivation and adherence.	“I’m naturally competitive, I want to hit my steps; if I’m close, I can’t stop trying [P34].”	19/47 (40%)
	4. Financial Incentivization	Rewards and insurance programs motivate health behaviors.	“UnitedHealthcare Motion connects to my watch; set goals and rack up money in a savings bank; see rewards for goals met [P10].”	11/47 (23%)
	5.Identity Protection	Anonymity or distance makes disclosure safer for some.	“Not face to face; it’s easier to talk; he doesn’t know who I am, just phone calls, so I feel more at ease [P16].”	3/47 (6%)

Themes by domain with brief definitions, exemplar quotations, and prevalence (*N* refers to the number of individuals endorsed).

#### Traditional healthcare barriers

Five barriers in traditional healthcare settings emerged: Financial Access Constraints, Structural System Limitations, Provider Bias and Discrimination, Cultural Belief Barriers, and Pandemic-Related Disruptions.

1. Cultural Belief Barriers (94%) emerged as the most endorsed traditional healthcare barrier. This theme captured how cultural norms and family dynamics influenced the recognition and discussion of health conditions. Participants described how cultural and regional traditions shaped communication about health concerns, often limiting open discussion of certain conditions. As one participant explained: “*Because my parents were from South Carolina, you didn’t go around talking …; you kept everything to yourself … so I’ve suppressed a lot of stuff* [P16, age 56, non-Hispanic multiracial, middle income].” This testimony illustrates how regional cultural norms around health communication can be transmitted intergenerationally, creating patterns of health information suppression that may delay or prevent healthcare seeking.

2. Financial Access Constraints (89% of participants) captured how personal and family financial circumstances directly affected healthcare access and decision-making, including difficult trade-offs between healthcare needs and other essential expenses. Participants frequently described delaying or avoiding care due to costs, struggling with insurance limitations, and experiencing stress related to medical expenses: “*Sometimes I think … I should really go to a therapist …, but … God they’re so expensive … I could put that money towards like different things* [P28, age 39, Black, high income].” These financial barriers affected participants across income levels, with 96% of low-income participants (*n* = 23/24), 85% of middle-income participants (*n* = 11/13), and 78% of high-income participants (*n* = 7/9) reporting cost-related concerns, suggesting that cost considerations influence healthcare decisions regardless of financial resources.

3. Structural System Limitations (79% of participants) captured organizational impediments within the healthcare system that create or perpetuate access disparities distinct from individual financial constraints. These barriers included organizational inefficiencies, administrative complexities, and systemic workforce shortages leading to provider unavailability: *“It’s very frustrating to try to find a PCP [primary care provider] … I waited a month and a half … I think a lot of people struggle with is being able to make appointments to see doctors in a timely manner …* [P36, age 34, Hispanic White, middle income]*.”*

This reflection highlights how institutional barriers, such as provider shortages and inefficient scheduling systems, can limit access to care even for those with sufficient financial resources, pointing to systemic issues embedded within the healthcare infrastructure itself.

4. Provider Bias and Discrimination (74% of participants) captured encounters with inequitable treatment in healthcare settings. Participants described patterns of being dismissed, subjected to inadequate testing, or receiving lower-quality care—experiences disproportionately affecting marginalized groups. These concerns were often deeply entwined with racial and ethnic identity, amplifying barriers to trust and access. As one participant explained: *“When it comes to Black-African American women … [providers] … don’t go as far as the extent [to] helping them … my son goes to the doctor, he sees the shot … and [cries]. “Oh, what’s wrong with him? He has a behavior problem?” No, he don’t like doctors* [P42, age 42, Black woman, low income].” This participant described a moment in which her child’s distress was pathologized rather than understood. Moments like these erode trust and reinforce feelings of being misunderstood and mistreated by the healthcare system.

5. Lastly, Pandemic-related Disruptions (60% of participants) highlighted how COVID-19 intensified existing healthcare disparities through increased stressors and reduced access to care. Participants described how the pandemic simultaneously limited healthcare availability while heightening health needs and eliminating crucial support resources, such as childcare, compounding challenges: *“I’ve dealt with depression on and off my entire life and I’ve got that under control, but … I just feel like in the last year … my mental health has started to physically affect my body … I wasn’t speaking to [my counselor] a lot because … I was with my kid … I didn’t have time and … childcare* [P34, age 43, non-Hispanic white, high income]”. The prevalence of these experiences suggests the pandemic created unique structural challenges rather than simply exacerbating existing healthcare barriers.

#### Digital health technology barriers

Four themes emerged as barriers to DHT utilization: Relational Connection Loss, Digital Literacy and Access Gaps, Data Security Concerns, and Value-Cost Assessment.

1. Relational Connection Loss (72% of participants) centered on concerns about diminished interpersonal elements of healthcare when delivered through digital platforms. Participants described how virtual platforms could not replicate the nuanced dynamics of face-to-face healthcare encounters, potentially compromising care quality and therapeutic relationships: “*I prefer mental health in person, especially for [child’s name] … I could deal with it, but I prefer the human connection* [P18, age 37, Hispanic, middle income].” This sentiment reflected a multifaceted concern about digital healthcare delivery, including difficulties in establishing therapeutic rapport, challenges in maintaining engagement without in-person accountability, and limitations in remote assessment, particularly for children and those with physical conditions. The prevalence of this concern across demographic categories suggests that preserving human connection represents a core challenge for digital health implementation, regardless of technological proficiency or healthcare literacy. This pattern held across income levels (67% of low-income, 62% of middle-income, and 100% of high-income participants), education levels (40% of those with high school education or less, 64% of those with some college, and 82% of those with bachelor’s degrees or higher), age groups (68% of those under 40, 77% of those 40-50, and 50% of those over 50), and racial/ethnic groups (73% of Hispanic/Latino, 60% of Black/African American, and 100% of White participants).

2. Digital Literacy and Access Gaps (45% of participants) captured challenges of technical proficiency and technology access that can impede effective utilization of digital health resources. These barriers showed interesting patterns across educational levels, with 57% of participants with some college education experiencing these gaps, compared to 40% of those with low education and 39% of those with higher education, suggesting that digital literacy challenges may not follow traditional educational hierarchies. The challenges often extended to family members who become de facto technical support: *“My mom … trying to get her on … Zoom … I have to walk her through like every step … for people that are not tech savvy, it has been a little bit of a barrier for them … to access … the portals* [P28, age 39, Black, middle income].”This quote illustrates how digital literacy barriers can create an additional burden on family members and potentially compromise healthcare independence.

3. Data Security Concerns (36% of participants) encompassed apprehensions about data protection and digital security that influence users’ willingness to engage with DHTs. “*The only other concern would be hackers, but I’m sure they’ll find a way around it* [P37, age 30, Black, low income].” This statement conveys a sense of resignation, acknowledging data breaches as an unavoidable feature of digital life, which may lead users to approach digital health tools with caution or reluctance.

4. Lastly, Value-Cost Assessment (28% of participants) reflected both financial constraints and attitudes about the perceived value of investing in digital health tools. Unlike traditional healthcare barriers where financial factors primarily affected access to care, in the digital context, these factors influenced both the ability to access technology and willingness to invest in digital health solutions: “*To really get the meat from [the app], you have to pay … Some [apps] are really expensive. I would be into them if they were covered by insurance* [P3, age, 43, Hispanic White, low income].” This reflection illustrates how the intersection of cost barriers and value perceptions shapes engagement with digital health tools, presenting a challenge distinct from traditional healthcare cost barriers. Although traditional healthcare costs were typically viewed as essential, digital health tools were subject to additional scrutiny, with participants questioning whether their benefits justified the expense.

#### Traditional healthcare facilitators

Although participants faced significant barriers in traditional healthcare settings, they also identified crucial facilitating factors that aided their healthcare navigation. Our analysis revealed four key facilitators: Supportive Relationship Networks, Healthcare Knowledge Brokers, Cultural Resource Integration, and Non-Clinical Digital Resources.

1. Supportive Relationship Networks (94% of participants) underscored the essential role of close personal ties in navigating healthcare. These networks, comprising family, friends, and partners, offered more than just emotional support. They functioned as relational safety nets, offering encouragement, helping interpret medical information, and guiding participants through logistical hurdles in the healthcare system. The support did not necessarily depend on formal medical expertise but on trust, availability, and lived experience. As one participant described: “*I do have a good network. My older sister’s a social worker… and [tells] me, ‘Hey listen, go visit this doctor’* [P26, age 39, Hispanic, low income].” This quote illustrates how emotional and practical support often overlap in everyday caregiving relationships, reinforcing participants’ ability to access and act on healthcare needs.

2. Healthcare Knowledge Brokers (77% of participants) referred to a specific subset of support relationships—those in which the person providing help had formal training or insider knowledge of the medical system. These individuals, often family members or friends with clinical or administrative expertise, served as informal healthcare consultants. Their guidance often shaped how participants interpreted symptoms, navigated diagnoses, or sought specialized care. For example: “*My aunt is a psychiatrist. She did all her testing on me when I was a young kid, so it’s always been part of my life—you need help, you ask for it* [P10, age 43, Multiracial, middle income].” These brokers were especially powerful facilitators because they bridged the gap between layperson and professional healthcare systems, providing tailored guidance grounded in medical knowledge.

3. Cultural Resource Integration (55% of participants) reflected the crucial role of cultural traditions, spiritual practices, and community values in shaping healthcare decisions and treatment preferences. For many, these resources served not only as complements to formal healthcare systems, but as vital sources of emotional support, caregiving, and healing: *“I have people that go to my church … help me with my family, … give my children love … in times … I don’t give my children the love that they deserve … They’ll get love from, you know, people in my church … I have a huge village of people that’s willing to help me and I love it … I [just] have to ask for help* [P33, age 37, Black, Low income].” This narrative highlights how faith communities operate as extended support systems, offering resources and care beyond those typically available in healthcare settings. It also illustrates how cultural values shape not only what types of support are available but also the norms surrounding help-seeking behavior, such as the emphasis on communal responsibility, the personal challenge of asking for assistance, and the behavioral norms around accessing them.

4. Lastly, Non-clinical Digital Resources (23% of participants) reflected the use of general online platforms, such as social media, forums, and search engines, as tools for navigating traditional healthcare systems. These platforms did not provide direct care but served as crucial intermediaries for locating providers, researching treatments, and connecting with others facing similar challenges: “*I’m not even exaggerating when I say this, I’m buying insulin off of other people online* … *there’s a big network right now on Facebook, actually, of people who buy even used insulin* … [P9, age 47, Hispanic White, high income].” This account highlights how, in the absence of accessible formal resources, individuals turn to informal digital networks to meet pressing medical needs. These platforms function not only as information hubs but also as makeshift support systems, filling gaps when traditional channels fall short.

#### Digital healthcare facilitators

Five themes emerged as facilitators of DHT use, reflecting patterns in participants’ narratives that diverged from traditional facilitators in both forms and their relationship to social identity. These included: Accessibility and Flexibility, Personalized Health Management, Financial Incentivization, Engagement Mechanisms, and Identity Protection.

1. Accessibility and Flexibility (96% of participants) was the most frequently cited digital facilitator, with many highlighting how digital health tools help reduce traditional barriers related to time, distance, and scheduling: *“I didn’t like telehealth before, but now… I like it because I can sit in my living room at 7:15, do my kid’s doctor appointment and then they can go to school … For a check in, it’s just made my life so much easier … My perspective has completely changed about technology* [P29, age 35, Black, middle income].” This example illustrates how digital tools can meaningfully alleviate logistical challenges in traditional care by offering greater scheduling flexibility and convenience.

2. Personalized Health Management (57% of participants) captured how digital tools enable customized health monitoring and intervention. Participants described how these tools could be adapted to track various aspects of their health journey simultaneously: “*It gives you ideas about how to think, mindfulness … do the breath app with the Apple Watch … going on the treadmill or outside for a walk or any type of exercise you can link it* [P2, Non-Hispanic White, Low income].” Unlike traditional healthcare’s episodic nature, digital tools offered continuous, personalized health tracking and feedback tailored to individual needs and preferences.

3. Engagement Mechanisms (40% of participants) encompassed features that promote consistent healthcare engagement through gamification, goal setting, and performance tracking. Features like streak tracking, rewards, and leaderboards tapped into users’ motivation for achievement and competition, encouraging engagement. As one participant explained: *“I’m naturally competitive … I want to get to my steps in my goal, and … if I get close to it, then it’s like I can’t stop … like today I’ll have … 1000 … I won’t even try because like I’m nowhere near it … If I was at four or five then I’d be like trying* [P34, age 43, Non-Hispanic White, High income].” This quote illustrates how gamified elements can spark engagement by activating a threshold of perceived attainability—users are more likely to persist when goals feel within reach. These patterns underscore the need for thoughtfully designed feedback to sustain user motivation over time.

4. Financial Incentivization (23% of participants) reflected the perceived economic advantages of digital health tools, particularly when linked to insurance benefits or reward systems. In contrast to traditional healthcare’s often opaque and unpredictable costs, digital tools were seen as more transparent and financially motivating: “*I use Apple Fitness and it’s called United Healthcare Emotion. It’s like an app for my health insurance and it connects to my watch, … set goals … [and has] a savings bank … you rack up like money in this bank. So, I can see the rewards of different goals that I meet…* [P10, age 43, Hispanic, middle income].” This example highlights how integrating digital health tools with financial incentives can reinforce health behaviors by making progress visible and materially rewarding.

5. Lastly, while endorsed by only a small subset of participants (6%), Identity Protection highlighted how digital interfaces can facilitate the disclosure of sensitive health information by reducing face-to-face anxiety and perceived judgment: *“The digital health tools might help because … you’re not actually sitting there face to face … I was just telling my counselor it’s a lot easier to [talk] to him … he doesn’t know who I am, he’s never seen me, [its] just phone calls, and so I feel more at ease talking to him…* [P16, age 56, non-Hispanic multiracial, middle income].” Though infrequent, participants said physical distance sometimes fostered openness; for some, digital tools may ease disclosure about stigmatized conditions or anxiety-provoking encounters.

#### Comparing traditional and digital healthcare barriers and facilitators

##### Traditional and digital barriers comparison

**Fig. 1:**
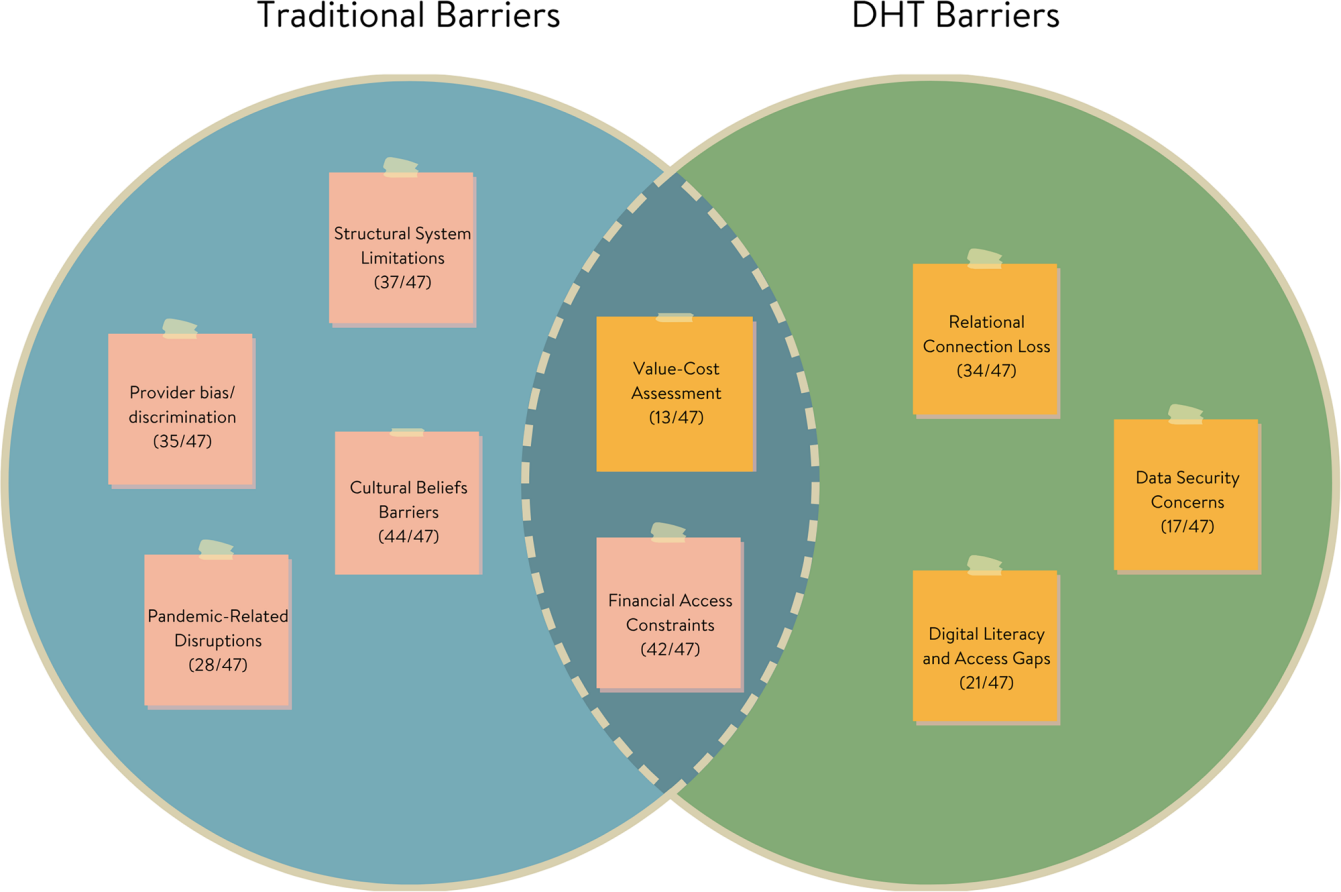
Comparison of barriers across traditional and digital healthcare contexts. Visual representation showing how barriers transform from traditional to digital healthcare settings, with overlapping and distinct elements highlighted.

Healthcare barriers shifted in form, prevalence, or salience as participants considered traditional versus digital healthcare contexts (Fig. 1). Of the eight total barrier themes identified, one persisted across both settings, four were specific to traditional care and did not surface in digital contexts, and three were specific to digital contexts. Financial Access Constraints was the only barrier that persisted across both traditional and digital contexts, though it decreased in endorsement and shifted in its expression. In traditional settings (endorsed by 89% of participants), it referred to out-of-pocket costs and insurance gaps, whereas in digital contexts (28%), it focused more on technology costs and subscription fees, as well as their perceived value relative to their benefits.

By contrast, four barriers commonly cited in traditional care were not reported in digital contexts. These included Structural System Limitations (79%), such as difficulty scheduling or navigating clinic bureaucracy; Provider Bias and Discrimination (74%); Pandemic-related Disruptions (60%); and Cultural Belief Barriers (94%), which encompassed stigma, mistrust of formal systems, and preference for culturally grounded care. Although these themes were absent as standalone barriers in the digital context, some cultural influences may have been indirectly reflected in digital themes, especially through participants’ hesitancy to pay for app-based care or their discomfort with unfamiliar digital models. This overlap suggests that certain traditional barriers may not disappear but instead become integrated into the framing of new digital concerns.

At the same time, three new barriers emerged exclusively in digital contexts. Relational Connection Loss (72%) captured how the absence of face-to-face interaction in telehealth and app-based care diminished emotional rapport, connection, or trust. Digital literacy and Access gaps (45%) surfaced as a key challenge, reflecting disparities in technological comfort, device access, and internet connectivity. Finally, Data Security Concerns (36%) reflected participants’ fears about privacy, surveillance, and digital vulnerability, which were unique to the digital format.

##### Traditional and digital facilitator comparison

**Fig. 2:**
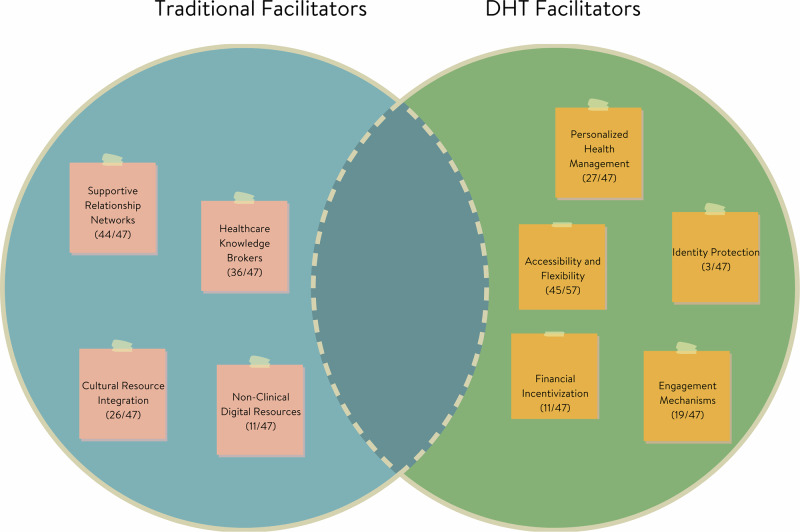
Comparison of facilitators across traditional and digital healthcare contexts. Visual representation illustrating the distinct nature of facilitators in traditional versus digital healthcare settings, showing minimal overlap between domains.

Facilitators of healthcare access and engagement also shifted across traditional and digital health contexts (Fig. 2). Of the nine total facilitator themes, none were shared across both contexts in an identical form, though one showed conceptual overlap. Four were unique to traditional care, and five emerged specifically in digital contexts, reflecting how evolving healthcare modalities give rise to new support mechanisms while phasing out others.

In traditional healthcare, participants emphasized interpersonal and community-based facilitators. These included Supportive Relationship Networks (94%), such as family or friends who offered emotional support and help navigating care; Healthcare Knowledge Brokers (77%), referring to support figures with medical expertise; Cultural Resource Integration (55%), including faith-based and cultural community supports; and Non-Clinical Digital Resources (23%), such as using social media or forums to locate care. These facilitators underscored the relational and contextual nature of traditional healthcare access, particularly for communities that rely on informal systems of trust and guidance.

Digital contexts introduced an entirely different set of facilitators shaped by the nature of technology. Accessibility and Flexibility (96%) was the most endorsed digital facilitator, reflecting how digital health tools removed barriers of time, transportation, and scheduling. Participants also valued Personalized Health Management (57%), including goal-setting and self-monitoring features; Engagement Mechanisms (40%), such as gamification and feedback loops; Financial Incentivization (23%), which linked health behaviors to rewards or insurance benefits; and Identity Protection (6%), with some participants feeling more comfortable disclosing health issues in private, digital spaces.

### Quantitative analysis

**Fig. 3:**
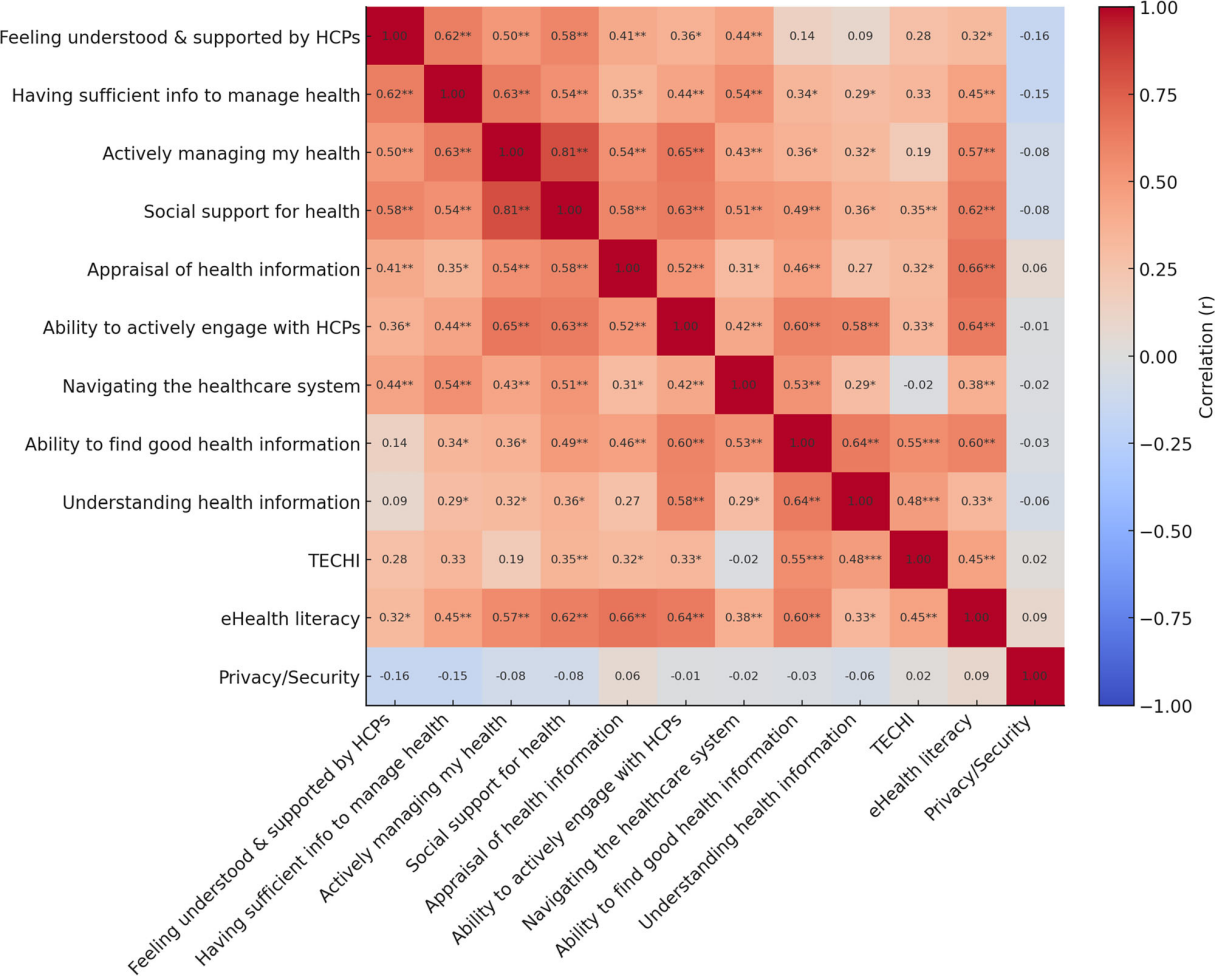
Spearman correlation matrix across health literacy, technology experience, eHealth literacy, and privacy and security variables. Values represent Spearman’s rank-order correlation coefficients, with *p < 0.005, **p < 0.01, ***p < 0.001. Variables reflect subscales of the Health Literacy Questionnaire (HLQ); the Technology Experiences and Challenges Inventory (TECHI Total); eHealth Literacy Scale (eHealth Literacy Total); and composite Privacy and Security Total scores. Correlation heatmap generated using ChatGPT (OpenAI, 2024) and formatted in Microsoft PowerPoint.

Quantitative analyses revealed notable interrelationships among the health literacy domains (Fig. 3). Social Support demonstrated strong positive associations with both Active Management (*r*_s_ = 0.81, 95% CI [0.66, 0.90], *p* < 0.001) and Engagement with Providers (*r*_s_ = 0.63, 95% CI [0.40, 0.79], *p* < 0.001), highlighting the interconnected nature of relational and functional health literacy. eHealth literacy functioned as a bridge between traditional and digital competencies, showing moderate correlation with Technological Proficiency (*r*_s_ = 0.40, 95% CI [0.11, .62], *p* = 0.006) and strong correlation with Social Support (*r*_s_ = 0.62, 95% CI [0.37, 0.78], *p* < 0.001). Notably, Privacy and Security Concerns were not significantly correlated with any other measured variables (all *ps* > 0.05), suggesting that concerns about digital privacy may represent a distinct dimension of digital health engagement. Descriptive statistics appear in Table 3; full correlations and CIs are in Tables 2 and 3.

**Table 3:** Descriptive statistics for quantitative variables

Measures	*M* (SD)	Range
‘Feeling understood and supported by healthcare providers’	13.23 (3.01)	4–16
‘Having sufficient information to manage health’	12.37 (2.47)	8–16
‘Actively managing health’	14.53 (2.80)	8–20
‘Social Support for health’	15.86 (2.67)	11–20
‘Appraisal of health information’	14.81 (2.74)	8–20
‘Ability to actively engage with healthcare providers’	20.83 (3.66)	9–25
‘Navigating the healthcare system’	24.09 (4.03)	16–30
‘Ability to find good health information’	19.55 (4.54)	10–25
‘Understanding health information well enough to know what to do’	22.87 (2.35)	18–25
e-Health Literacy Scale	33.79 (5.15)	19–40
Attitudes towards Mobile Privacy and Security	8.85 (2.47)	3–13
Technological Ease and Computer-Based Habits Inventory	65.40 (10.10)	47–85

*M* mean, *SD* standard deviation; range indicates minimum to maximum scores.

### Integration of qualitative and quantitative analysis

**Fig. 4:**
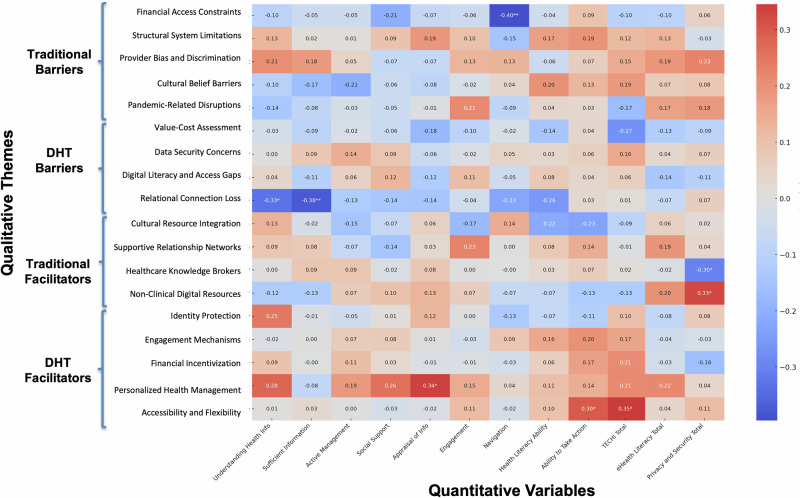
Spearman correlations between qualitative themes and quantitative measures of health literacy, technology experience, eHealth literacy, and privacy and security variables. Values represent Spearman’s rank-order correlation coefficients, with *p < 0.05, **p < 0.01, ***p < 0.001. Quantitative measures include subscales from the Health Literacy Questionnaire (HLQ; Understanding Health Info, Sufficient Information, Active Management, Social Support, Appraisal of Info, Engagement, Navigation, Health Literacy Ability, Ability to Take Action, and HLQ Total), the Technology Experiences and Challenges Inventory (TECHI Total), the eHealth Literacy Scale (eHealth Literacy Total), and composite Privacy and Security Total scores. Qualitative themes reflect sociocultural, technological, and individual-level factors identified through thematic analysis of participant interviews. Correlation heatmap generated using ChatGPT (OpenAI, 2024) and formatted in Microsoft PowerPoint.

Integration enabled examination of how key qualitative themes mapped onto individual differences in health and digital competencies. Spearman’s rank-order correlations between code frequencies (i.e., narrative salience) and quantitative measures highlighted several core qualitative insights (Fig. 4; Supplementary Tables 4 and 5). Within traditional healthcare barriers, Financial Access Constraints were negatively associated with navigating the healthcare system (*r*_s_ = −0.40, 95% CI [−0.62, −0.11], *p* = 0.006), supporting participants’ narratives that financial limitations restrict not just access but the ability to act on health information. In the DHT barrier domain, Relational Connection Loss showed significant negative associations with having sufficient information to manage health (*r*_s_ = −0.38, 95% CI [ −0.61, −0.09], *p* = 0.009) and feeling understood and supported by providers (*r*_s_ = −0.33, 95% CI [−0.57, −0.04], *p* = 0.024). These patterns align with participant concerns that digital platforms can dilute communication and clarity in ways that interfere with comprehension and trust.

Several correlations also differentiated how traditional and digital facilitators operate. Reliance on Healthcare Knowledge Brokers (i.e., trusted individuals with medical expertise) was negatively correlated with privacy/security concerns (*r*_s_ = −.30, 95% CI [−0.54, −0.01], *p* = .039), suggesting that interpersonal guidance may heighten awareness of data-security risks. In contrast, use of Non-Clinical Digital Resources (e.g., search engines) was positively associated with privacy/security concerns (*r*_s_ = 0.33, 95% CI [0.04, 0.57], *p* = 0.025), potentially reflecting greater sensitivity among heavier information seekers. Within digital facilitators, Personalized Health Management correlated with appraisal of health information (*r*_s_ = 0.34, 95% CI [0.05, 0.57], *p* = 0.021), consistent with reports that digital tools feel most empowering when users are confident evaluating health information. Additionally, Accessibility and Flexibility were positively associated with understanding health information well enough to know what to do (*r*_s_ = 0.30, 95% CI [0.01, 0.54], *p* = 0.042) and with technological proficiency (*r*_s_ = 0.35, 95% CI [0.06, 0.58], *p* = 0.017), reinforcing qualitative accounts of how DHTs remove logistical barriers and promote engagement.

### Social identity intersections

Because identity can shape experiences even when it is not explicitly named, we report two complementary signals: narrative salience (explicit identity mentions co-occurring with themes) and measured identity–theme associations (participant-level theme presence/absence correlated with recorded demographics). Using this two-signal approach, identity dynamics were voiced more often in traditional care narratives and appeared more subtly in digital contexts.

### Co-occurrence analysis

Qualitative co-occurrence analysis indicated that participants referenced their social identities far more frequently when discussing traditional healthcare settings, with 94 identity-barrier/facilitator intersections coded across the transcripts. Cultural Belief Barriers were the most frequent site of identity intersection, especially with religious/spiritual identity (8 co-occurrences), race/ethnicity (6), and family roles (5). Structural System Limitations and Supportive Relationship Networks also commonly intersect with race/ethnicity, income level, and religious identity. In contrast, identity-related co-occurrences were far less common in digital healthcare narratives, with only 9 coded intersections across the dataset. Where they did appear, identity was linked with practical challenges, such as income intersecting with Affordability Concerns (3 co-occurrences) or education level intersecting with Convenience and Access (2).

### Correlational patterns

**Fig. 5:**
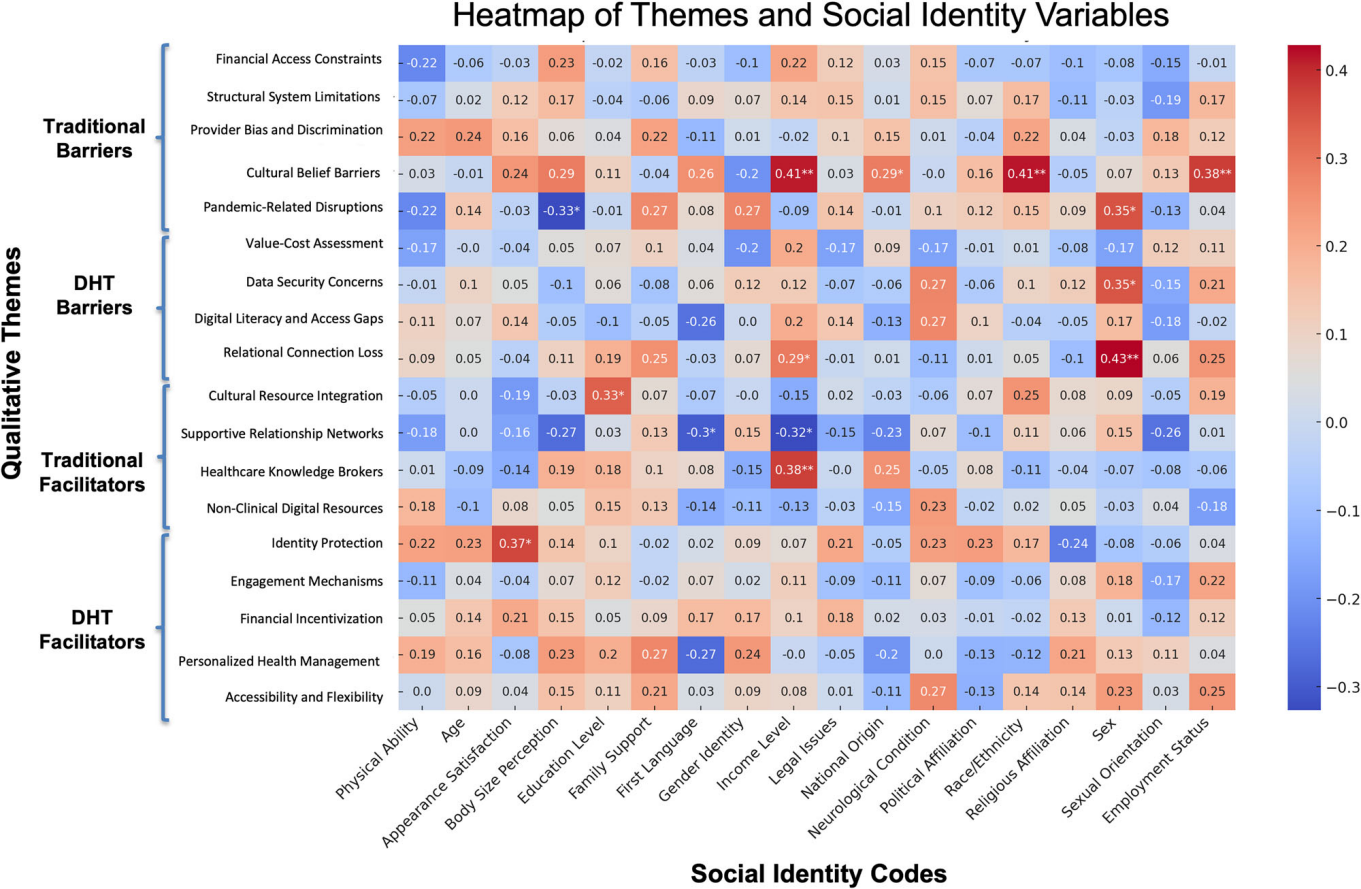
Spearman correlations between qualitative themes and participant social identity codes. Heatmap showing Spearman correlations between mentions of social identity and identified themes, with *p < 0.05, **p < 0.01, ***p < 0.001. Social identity codes reflect participant-reported demographic characteristics that were coded, including physical ability, age, appearance satisfaction, body size perception, education level, family support, first language, gender identity, income level, legal issues, national origin, neurological condition, political affiliation, race/ethnicity, religious affiliation, sex, sexual orientation, and employment status. Qualitative themes include Traditional Barriers (e.g., financial access constraints, structural system limitations, provider bias), DHT Barriers (e.g., data security concerns, digital access gaps), Traditional Facilitators (e.g., healthcare knowledge brokers, cultural resources), and DHT Facilitators (e.g., engagement mechanisms, personalization, accessibility). Correlation heatmap generated using ChatGPT (OpenAI, 2024) and formatted in Microsoft PowerPoint.

Although identity references were more commonly voiced in traditional care narratives, correlational analyses revealed measurable associations between social identity variables and specific barriers and facilitators in both traditional and digital settings (Fig. 5; Supplementary Tables 6 and 7). In traditional healthcare, participants who referenced race/ethnicity or income were significantly more likely also to discuss Cultural Belief Barriers (race/ethnicity: *r*_s_ = 0.41, 95% CI [0.13, 0.63], *p* = 0.004; income: *r*_s_ = 0.41, 95% CI [0.13, 0.63], *p* = 0.004), as were those who referenced employment status (*r*_s_ = 0.38, 95% CI [0.09, 0.61], *p* = .008). Mentions of national origin were also positively associated with Cultural Belief Barriers (*r*_s_ = 0.29, 95% CI [− 0.01, 0.54], *p* = 0.049). Participants who referenced their sexual orientation were more likely to describe Pandemic-Related Disruptions (*r*_s_ = 0.35, 95% CI [0.06, 0.59], *p* = 0.016), while those who referenced their body size were less likely to do so (*r*_s_ = −0.33, 95% CI [−0.57, −0.04], *p* = .025). Income mentions were associated with discussion of Healthcare Knowledge Brokers (*r*_s_ = 0.38, 95% CI [0.09, 0.61], *p* = 0.009), and references to education were positively correlated with Cultural Resource Integration (*r*_s_ = 0.33, 95% CI [0.04, 0.57], *p* = 0.023).

In digital contexts, references to participants’ social identities continued to align with distinct themes in their healthcare narratives. Participants who mentioned their sexual orientation were also more likely to describe Relational Connection Loss (*r*_s_ = 0.43, 95% CI [0.15, 0.65], *p* = 0.003) and Data Security Concerns (*r*_s_ = 0.35, 95% CI [0.06, 0.59], *p* = 0.017), suggesting that identity-related considerations may shape concerns about trust, privacy, and interpersonal dynamics in digital settings. Mentions of first language and income level were negatively associated with Supportive Relationship Networks (first language: *r*_s_ = −0.30, 95% CI [−0.54, −0.01], *p* = 0.042; income: *r*_s_ = −0.33, 95% CI [−0.57, −0.03], *p* = 0.026), indicating that participants who referenced these identity markers may have been more likely to report challenges in accessing meaningful support. Participants who mentioned income were also more likely to discuss Relational Connection Loss (*r*_s_ = 0.29, 95% CI [−0.00, 0.54], *p* = 0.050), while references to appearance satisfaction were associated with greater discussion of Identity Protection (*r*_s_ = 0.37, 95% CI [0.08, 0.60], *p* = 0.011).

Several notable demographic patterns emerged (see Table 4 for significant *p*-values and Supplementary Tables 8 and 9 for complete analyses). Cultural Belief Barriers were more likely among full-time employed participants and those reporting languages beyond English/Spanish. Relational Connection Loss showed socioeconomic gradients, being less common among low-income participants but more common among high-income and highly educated participants. Data Security Concerns were higher among U.S.-born participants and those with higher education. Accessibility and Flexibility decreased with age, while Identity Protection increased with age. These patterns suggest that social positions shape both the salience of different barriers and facilitators and participants’ strategies for navigating traditional versus digital healthcare contexts.

**Table 4:** Significant Spearman rank-order correlations between barrier/facilitator themes and demographic variables

Theme	Demographic variable	*rs*	95% CI	*p*
	Traditional healthcare barriers			
Cultural belief barriers	Full-time employment	0.42	[0.14, 0.64]	0.003
	Homemaker	−0.49	[−0.69, −0.21]	<0.001
	Spoken Language—Other‡	0.40	[0.12, 0.62]	0.06
Provider discrimination	Retired	NC†	-	-
COVID-19 disruptions	Age	−0.34	[−0.58, −0.05]	0.02
DHT barriers				
Relational connection loss	Low income	−0.31	[−0.55, −0.01]	0.04
	High income	0.43	[0.15, 0.65]	0.003
	Mid education	−0.29	[−0.54, −0.00]	0.05
	High education	0.37	[0.08, 0.60]	0.01
Data security concerns	Age	−0.29	[−0.54, −0.00]	0.05
	Born in USA	0.31	[0.02, 0.55]	0.034
Value-cost assessment	High education	0.36	[0.07, 0.59]	0.01
	Spoken Language—Other	0.31	[0.02, 0.55]	0.03
Digital literacy and access	Spoken Language—Spanish	−0.31	[−0.55, −0.02]	0.01
Traditional healthcare facilitators				
Supportive relationships	Part-time employment	−0.43	[−0.64, −0.15]	0.003
	Black/African American	0.32	[0.03, 0.56]	0.03
Cultural resource integration	Low income	−0.33	[−0.58, −0.04]	0.02
Digital health facilitators				
Accessibility/flexibility	Age	−0.32	[−0.56, −0.03]	0.03
Personalized health management	Spoken Language—English	0.32	[0.03, 0.56]	0.03
Financial incentivization	Different sex orientation	NC†	-	-
	Bisexual	NC†	-	-
Identity protection	Age	0.35	[0.06, 0.59]	0.02
	American Indian/Alaska Native	NC†	-	-
	Retired	NC†	-	-

Associations are between participant-level theme presence (0/1) and a recorded demographic variable; *N* = 47. Only effects with *p* < 0.05 are shown (except sparse rows, marked †). CIs use Bonett–Wright (Fisher *r*-to-*z*). †Sparse category (*n* < 5); statistics not computed, reported descriptively only (NC). FDR (Benjamini–Hochberg) applied across non-sparse tests. Demographics were dummy-coded (age continuous). ‡ “Other language(s) spoken” indicates speaking languages beyond English/Spanish.

## Discussion

This convergent mixed-methods investigation examined how female caregivers during COVID-19 perceived differences in barriers to healthcare access between traditional and digital contexts. Participants describe insurance-related costs in traditional contexts and technology-related expenses in digital contexts, highlighting how disparities can be maintained through new forms, even as delivery models change. Although participants described these barriers less frequently in digital settings, they did not disappear; instead, they were described more often as technology costs, challenging the notion that digital health consistently lowers financial barriers. Rather than expanding access for underserved communities, DHTs may recreate exclusions in different ways, such as device requirements, data costs, or app subscriptions. Furthermore, participants reported novel digital-specific barriers, including relational disconnection, gaps in digital literacy and access, and privacy concerns, which underscores how technological solutions can simultaneously reduce traditional obstacles while introducing new ones^44^.

This pattern also extended to facilitators. While relationship-based supports are documented as critical for marginalized populations^33,34^, participants described engagement strategies and feedback mechanisms in digital contexts that often require baseline digital fluency^45^. Prior work has shown that without these fundamental skills, digital health tools risk widening rather than reducing gaps in engagement, since effective use depends not only on access but also on the ability to navigate and apply electronic health information^45^. Participants highlighted this shift by describing how family guidance, trusted advice from knowledgeable community members, and faith-based support facilitated navigation in traditional care, whereas digital contexts relied more on self-tracking, gamified features, and insurance-linked rewards.

Importantly, our demographic analyses revealed that these patterns varied systematically across social positions. Cultural belief barriers were more prevalent among full-time employed participants and those speaking languages beyond English and Spanish, while concerns about relational connection loss showed clear socioeconomic gradients—being less common among low-income participants but more frequent among those with higher income and education. Data security concerns were notably higher among U.S.-born participants and those with higher education, while older participants were less likely to value digital accessibility but more likely to appreciate identity protection features. These patterns suggest that social positioning shapes not only healthcare experiences but also strategies for navigating both traditional and digital care contexts.

Crucially, identity-based disparities remained visible across both healthcare contexts, even as their expression shifted. While participants more frequently referenced their identities in relation to traditional healthcare, the quantitative and co-occurrence analyses revealed continued patterns in digital settings. In digital contexts, explicit identity references were less frequent but still consequential: for example, participants who referenced their sexual orientation described feeling greater relational disconnection and heightened concerns about data privacy, while those who referenced income or language reported fewer opportunities to access supportive digital networks. These findings align with broader patterns of digital inequality^27^ and suggest that digital tools may render disparities less visible, without addressing their root causes.

Taken together, participants’ accounts suggest that technology may not neutralize structural inequities but instead reconfigure how they are experienced. Traditional barriers such as financial access constraints, provider bias, and structural limitations reflect well-documented mechanisms of exclusion, particularly for marginalized populations^12,16,17^, and our study empirically demonstrated how these dynamics continue to shape care experiences in the present sample. Cultural belief barriers, endorsed by 94% of participants, further highlight the enduring influence of intergenerational norms and community dynamics on shaping care engagement. Replicating these findings in a diverse, contemporary cohort underscores their persistence and highlights the importance of addressing longstanding inequities in traditional care even as digital models expand, since both systems increasingly operate side by side. Because these tools are still in relatively early stages of development and adoption, there is an opportunity to identify and address these emerging challenges before they become entrenched features of the healthcare landscape.

At the same time, participants’ reports of digital-specific challenges point to new forms of exclusion. Digital literacy and access gaps (reported by 45% of participants) echo longstanding concerns about differential technology access^30,46^, while relational connection loss (endorsed by 72%) highlights how technology can disrupt the interpersonal foundations of care, especially among populations that rely heavily on trusted relationships and cultural concordance^18^. Participants frequently described frustration with the steep learning curve of digital platforms, noting how difficulties logging into portals or troubleshooting apps often required outside help and undermined their healthcare independence. Others expressed concerns that privacy breaches were inevitable, which discouraged them from fully engaging with digital tools even when access was available. Facilitators also shifted, from culturally embedded supports and healthcare knowledge brokers to digital engagement strategies and personalization features, structures that may advantage those with more technological resources and comfort.

These shifts suggest that DHTs were not perceived as neutral delivery channels. Participants described access as organized differently in ways that can perpetuate, and at times obscure, systemic inequities. For example, while traditional financial barriers often involved insurance gaps or copays, digital contexts introduced technology costs and app-based pricing structures—forms of exclusion that may be perceived as more voluntary or personal, but are equally tied to structural inequality^31^. The apparent absence of traditional barriers, such as provider discrimination or bureaucratic inefficiencies, in digital narratives could be misinterpreted as progress. However, the asynchronous, remote, and algorithm-driven nature of DHTs may make certain barriers less visible in reported accounts. This “invisibility” of disparities in digital settings may reflect reduced opportunities for discrimination to be directly observed, not its elimination. The shift from relationship-centered to technology-mediated facilitators is similarly consequential. Whereas traditional facilitators often leveraged social capital and cultural resources^33^, digital facilitators, such as those utilizing gamified features or algorithmic personalization, demand individual proficiency and sustained device engagement. The positive association between technological proficiency and perceived accessibility *(r*_s_ = 0.35, *p* < 0.05) suggests that DHTs may stratify access by digital skills, not just medical need. This has critical implications for equity; communities that previously relied on interpersonal networks may be left without comparable support in digital spaces. Without culturally grounded digital alternatives, the transition to DHTs risks privileging already-connected users while deepening divides for those with lower technological access, confidence, or trust.

The integration of quantitative findings further substantiates and enriches the thematic results. Correlations among health literacy domains, particularly the strong links between social support, active health management, and provider engagement (*r*_s_ = 0.81, *p* < 0.001), highlight the interconnected nature of relational and functional healthcare literacy^47^. These results are consistent with the value of interpersonal support as a foundation for navigating care effectively. eHealth literacy served as a conceptual and statistical bridge between traditional and digital competencies, correlating with both technological proficiency (*r*_s_ = 0.45, *p* < 0.01) and social support (*r*_s_ = 0.66, *p* < 0.001). Similarly, higher scores on the TECHI, which measures general technological comfort and frequency of use, were positively associated with perceived accessibility and engagement with digital tools. These findings align with prior models that position eHealth literacy as essential to meaningful digital health engagement^45^. In contrast, privacy and security concerns appeared largely independent of other measures, suggesting that apprehensions about data protection form a distinct dimension of digital vulnerability that cuts across skill levels and access. Several correlations mirrored specific qualitative themes. For example, financial access constraints were negatively associated with healthcare navigation ability (*r*_s_ = −0.41, *p* < 0.01), reinforcing participant narratives that economic hardship affects not only whether care is pursued, but how successfully individuals engage with systems. Relational connection loss in digital contexts was associated with lower health information comprehension (*r*_s_ = −0.33, *p* < 0.05), consistent with concerns that interpersonal disruptions may relate to lower reported understanding.

Our identity-based analyses provide further insight into how DHTs may obscure, but not erase, the influence of social identity on healthcare experiences. In traditional settings, participants frequently linked identity to care barriers and facilitators, particularly around race, religion, income, and family roles^42^. These intersections reflect longstanding evidence of structural discrimination and identity-based disparities in clinical contexts^16,17^. Specifically, prior systematic reviews confirm that healthcare professionals often hold implicit racial, ethnic, and other biases, at levels comparable to the general population, and these implicit attitudes are linked to disparities in patient–provider interactions, treatment decisions, and quality of care^16,17^. In digital healthcare narratives, however, identity was referenced less often and in more indirect ways. This reduction in explicit identity linkage may stem from the algorithmic, asynchronous nature of DHTs, where social cues and interpersonal interactions are minimized. Yet correlational analyses revealed that identity-based disparities remained. For example, participants referencing sexual orientation reported higher relational disconnection (*r*_s_ = 0.43, *p* < 0.01) and greater data security concerns (*r*_s_ = .35, *p* < .05). Similarly, references to income or language were associated with diminished access to supportive digital networks (*r*_s_ = −0.32 and −0.30, respectively). These patterns align with broader concerns that digital health systems may reinforce existing disparities. Prior research shows that while digital tools such as patient portals and health apps can enhance engagement, they also risk multiplying existing disparities rather than simply adding new ones, as unequal access to broadband and digital literacy continues to disadvantage certain groups^27^. Similarly, Obermeyer et al. (2019) demonstrated that a widely used healthcare prediction algorithm underestimated the needs of Black patients because it relied on health costs as a proxy for illness; at the same risk score, Black patients were considerably sicker than White patients, resulting in far fewer being referred for extra care^48^. The absence of overt discrimination does not mean equity has been achieved—it may simply mean it has become more difficult to identify and address.

These findings highlight key opportunities for advancing equity in digital health by addressing the specific barriers and facilitators identified across traditional and digital contexts among COVID-era female caregivers. Because findings are specific to this group and time period, we frame them as transferable guidance for similar caregiving contexts rather than population-wide prescriptions. First, DHTs should be developed using universal design principles and co-design methods that include diverse end-users to ensure cultural relevance and usability across demographic groups. This aligns with participatory design frameworks that have demonstrated success in improving health technology adoption and usability among underserved populations^49–51^. Second, hybrid care models that integrate the relational strengths of traditional care with the efficiency and scalability of digital tools may offer the best outcomes for engagement and equity. Research supports that such models can enhance care continuity, especially for populations with structural barriers to access^52,53^.

Third, equity-focused evaluation metrics should be embedded in digital platforms, along with safeguards such as algorithm audits and user testing among marginalized populations to detect and mitigate bias. These strategies reflect a growing consensus on the importance of fairness and transparency in healthcare AI systems^54,55^. Finally, tailored education and outreach efforts are essential, both to raise awareness about privacy risks among low-literacy users and to demystify digital tools for those with limited technological access or confidence. Such approaches are grounded in eHealth literacy models that emphasize the need for user-centered communication and digital empowerment^45,56^. These strategies reflect the multi-level prevention approach underlying our health disparities definition, where prevention occurs through coordinated action by policymakers, healthcare institutions, and community organizations rather than individual patients.

Since data collection, the healthcare landscape has undergone substantial changes that may amplify the relevance of these patterns. By 2025, telehealth will no longer be an emergency substitute but a standard component of care, with most insurers covering virtual visits. However, some temporary pandemic expansions have been retracted, shaped by reimbursement and regulatory decisions regarding coverage for virtual and audio-only visits, cross-state licensure flexibilities, and broadband and digital equity investments. Care is increasingly delivered through hybrid models that combine digital and in-person visits. Secure messaging allows patients and family members to participate remotely, while wearable devices enable continuous health monitoring and self-management, particularly for chronic conditions. AI-driven tools—from symptom checkers to personalized reminders and predictive algorithms—are increasingly embedded in these platforms, supporting decision-making, efficiency, and tailored care.

Despite these advances, structural inequities persist and may become less visible without becoming less consequential. Technology costs, gaps in digital literacy, and uneven trust in digital systems continue to shape who benefits from innovations. As AI adoption accelerates, there is a risk of amplifying disparities if safeguards are not implemented proactively. The patterns of barriers and facilitators identified in this study may inform current decisions about digital health integration, underscoring the need to embed equity considerations at every stage of design and implementation.

To reduce inequities in care, digital innovations should be developed in ways that sustain relational aspects of treatment, ensure usability for individuals with varying technological skills, and embed transparent approaches to data protection. Additionally, these innovations should be paired with health-system guardrails, including preserving low-barrier channels (telephone/SMS), subsidizing connectivity/devices, simplifying authentication, embedding human navigators during portal setup, and conducting routine equity audits of patient-facing algorithms and workflows to prevent benefits from clustering among digitally advantaged groups.

This study offers several notable strengths. Using a convergent mixed-methods approach, it goes beyond surface-level assumptions that digital technologies inherently increase access to care. Instead, it centers participants’ voices to examine how experiences with digital health tools differ from existing barriers and facilitators. By comparing themes across traditional and digital healthcare settings, the study identifies which barriers persist, which appear different, and which are newly reported, offering a nuanced understanding of caregiver perceptions during the evolving healthcare landscape. This work foregrounds social identity as a critical factor in healthcare experiences. By examining both co-occurrence patterns in narrative data and correlations between identity mentions and other measures, the study captures how identity intersects with access and engagement. Few digital health studies have systematically examined how identity-based disparities translate to digital platforms, with most focusing on single-axis measures, such as broadband access or general digital literacy^27,46^. The combination of thematic extraction, cross-context comparison, quantitative integration, and identity-based analysis enables a comprehensive exploration of digital health equity.

Several limitations warrant consideration. First, while the sample centered on historically underserved populations, data were collected exclusively from female caregivers. Gendered differences in caregiving responsibilities^57^, healthcare access^58^, and digital technology^59^ use may influence both engagement with DHTs and responses to study measures. Findings may not generalize to other groups, such as male caregivers or non-binary individuals, who may experience distinct healthcare dynamics. Expanding demographic representation remains an ongoing need in digital health equity research^56^. Second, data were collected during the COVID-19 pandemic, a period that rapidly reshaped healthcare delivery. While this context offered a unique lens into digital health changes, it may have introduced temporal effects unlikely to persist post-pandemic. Longitudinal research is needed to examine whether patterns observed here hold as hybrid care models stabilize^60^. These findings must be replicated in future samples with more diverse caregiving samples and outside of the pandemic context. Third, interviews were conducted remotely. While we offered a telephone alternative and, through the parent study, loaner devices/data plans, a remote-only design may still advantage households with stable connectivity^61,62^ and adequate private space^63^, which can limit transferability. Future work should incorporate in-person and other offline options to reduce this barrier.

Fourth, Spanish-language interviews were translated into English for analysis, which may have resulted in some loss of cultural or linguistic nuance despite professional translation services. Additionally, although our thematic coding captured whether participants mentioned aspects of their identity and the broad identity category (e.g., race, gender, sexual orientation), it did not capture the specific identity value (e.g., identifying as Black, Latina, or transgender). Our approach captured explicit references to identity but may have missed more implicit or structural forms of inequity embedded in language or digital design. Standard qualitative methods can under-detect these subtler dynamics, especially in digital contexts^64^. Critically, our finding that identity was referenced less frequently in digital contexts may reflect limitations in our explicit coding approach rather than indicating that identity becomes less relevant in digital healthcare. This methodological limitation means that structural and implicit forms of discrimination may persist in digital settings but remain invisible to retrospective self-report approaches. The apparent “invisibility” of disparities in digital contexts should not be interpreted as evidence of reduced inequality but rather as a call for more sophisticated detection methods. Additional research could integrate natural language processing to detect biased or exclusionary phrasing, critical discourse analysis to examine how power and marginalization are reflected in participants’ narratives, and inclusive user experience testing to reveal how specific interface choices may unintentionally disadvantage certain groups.

Furthermore, the questionnaire component is theory-guided yet exploratory; we therefore emphasize effect magnitudes (*r*_s_) and 95% CIs and convergence with qualitative findings. In particular, the three-item privacy composite, reliable but brief, cannot capture privacy’s multidimensionality, and our multi-instrument battery prioritizes breadth to triangulate distinct facets of digital health readiness; accordingly, results are hypothesis-generating and point to fuller instruments and a priori tests in larger, stratified samples. We recommend subsequent research employ fuller instruments like the Internet Users’ Information Privacy Concerns (IUIPC) scale with larger, stratified samples for confirmatory testing. Finally, our cross-sectional design examining retrospective participant reflections captures perceived differences between healthcare modalities rather than actual transformation processes, which would require longitudinal observation of individuals transitioning between care types. However, understanding how the same individuals perceive barriers and facilitators across different care contexts provides valuable insights into healthcare access experiences.

As healthcare continues to shift into the digital domain, this study offers a critical lens on how barriers and facilitators are not merely replaced but fundamentally experienced differently across contexts. Through a nuanced, mixed-methods approach, findings demonstrate that digital health tools may reduce certain logistical constraints but also introduce new challenges related to connection, privacy, and literacy, particularly for populations who have been historically marginalized in healthcare. Rather than assuming digital tools are inherently equitable, these findings underscore the need for intentional design, inclusive evaluation, and ongoing vigilance. By centering the experiences of historically marginalized groups and integrating both qualitative and quantitative insights, we can better understand how digital care is experienced differently and how to ensure it moves us toward systems that are not only innovative but also just, inclusive, and accessible. Taken together, these context-specific results highlight design and implementation considerations for similar caregiver populations, while underscoring the need to validate these patterns in broader, post-pandemic samples.

## Methods

### Study design

**Fig. 6:**
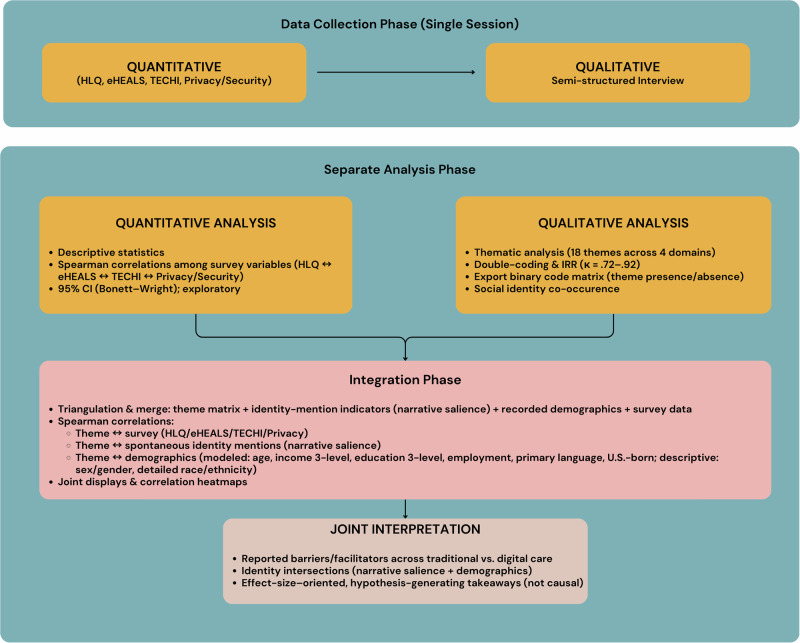
Convergent mixed-methods workflow. Surveys (HLQ, eHEALS, TECHI, Privacy/Security) were completed prior to a semi-structured interview in a single session. Quantitative and qualitative data were analyzed separately, then integrated by merging participant-level theme presence/absence with survey scores and demographics; identity intersections were examined via narrative salience (spontaneous identity mentions) and recorded demographics. Spearman correlations and joint displays (heatmaps) supported interpretation.

We used a convergent mixed-methods design with concurrent data collection in the same study visit (see *Procedure* for the within-visit order)^65^. Semi-structured interviews explored experiences with traditional and digital care, and structured surveys provided context on health literacy, eHealth literacy, technology use, and privacy/security concerns. Quantitative and qualitative data were analyzed separately and then integrated to identify convergence or divergence, supported by joint displays (e.g., correlation heatmaps). Reporting followed the Consolidated criteria for Reporting Qualitative Research (COREQ)^66^. Figure 6 summarizes the workflow from recruitment through separate analyses and integration.

### Participants

Forty-eight caregivers, drawn from a larger study examining family mental health (*N* = 56), opted for an in-depth study exploring their experiences with healthcare and DHTs (*M*age = 41.49, SD = 6.56, *range* = 26–56). One participant was excluded due to technical issues that prevented the collection of an adequate audio recording. Participants self-identified their racial/ethnic identity through two demographic questions: (1) Hispanic heritage (Hispanic/Latino/a, Non-Hispanic/Non-Latino/a, or Other, with open-text allowed); and (2) race (White, Black/African American, Asian, American Indian/Alaska Native, Native Hawaiian/Pacific Islander, Other, or Mixed race, with open-text allowed). Consistent with NIH guidance^67^, anyone identifying as a group other than non-Hispanic White was classified as underrepresented. The sample size is typical for thematic saturation^65,68^; however, given the modest sample size for quantitative analysis, we planned effect-size-oriented, hypothesis-generating checks rather than significance testing.

To better understand healthcare disparities among caregivers from diverse backgrounds, we established eligibility criteria focused on populations historically underserved in healthcare research. Caregivers of children aged 6–9 were included based on the design of the larger parent study, which focused on family and child mental health during early to middle childhood. This developmental window marks the start of formal schooling, introducing new academic and social demands that can influence children’s mental health trajectories and increase caregiver stress^69^. Recruitment targeted primary caregivers without gender restrictions, though the final sample reflected typical demographic patterns in family health research, where mothers serve as primary healthcare navigators.

To ensure the sample reflected families navigating meaningful contextual or mental health challenges, participants had to meet at least one of the following criteria: identifying as a member of an underrepresented racial-ethnic group (93.5% of sample met this criterion); having a child with elevated mental health symptoms defined as scoring at or above the 70^th^ percentile on at least one difficulty subscale of the Strengths and Difficulties Questionnaire (SDQ^70^; 100% of sample met criterion); or reporting household income at or below the 33rd percentile of their county, adjusted for family size (65.2% of sample met this criterion). The SDQ is a validated 25-item behavioral screening tool assessing emotional symptoms, conduct problems, hyperactivity/inattention, peer problems, and prosocial behavior. These inclusion criteria were chosen to capture families experiencing structural, socioeconomic, or psychological factors that may shape parenting and engagement with child mental health services without restricting the sample to a single clinical domain or dimension of adversity. English or Spanish language proficiency was also required to support meaningful participation and promote linguistic diversity in the sample. All inclusion criteria were guided by the American Psychological Association’s standards for inclusivity and bias-free language^71^. These inclusion criteria were designed to ensure representation of individuals most affected by structural healthcare inequities, aligning with the study’s intersectionality framework by examining how overlapping social identities shape healthcare experiences^40,41^.

### Procedure

Data collection occurred during the COVID-19 pandemic (2020-2023), a period that heightened existing healthcare disparities^72^ and accelerated the adoption of DHTs^60,73^. Participants were recruited via social media (Facebook/digital flyers), community/clinic referral, other online outreach, and personal referrals. Recruitment source was recorded for 32 participants ( ≈ 68%); among known sources, 71.9% were recruited through social media, 9.4% through clinic/community referral, 15.6% through other outreach, and 3.1% through personal referrals. Interested families completed a contact form followed by an eligibility screening. Eligible caregivers provided consent and, during the parent study’s baseline visit, were invited to enroll in this optional sub-study. Enrolled participants completed the sub-study in a single session with a fixed sequence: four self-report questionnaires (health literacy, eHealth literacy, technology use, and privacy concerns) followed by a semi-structured interview about healthcare and digital technology experiences. Participants received $40 compensation via gift card or Zelle. Interviews and questionnaires were linked at the individual level for integrated analysis. All procedures adhered to the Declaration of Helsinki ethical standards and were approved by the University IRB (IRB-20-011). Interview recordings and questionnaire data were de-identified and encrypted, with all study data stored in a secure, access-controlled database.

### Qualitative data collection

Semi-structured interviews lasting 45 to 50 min were conducted remotely, primarily via Zoom video. When video was not feasible, participants completed telephone interviews. For participants with device or connectivity barriers, loaner smartphones with temporary data plans were available through the parent study. The interview protocol was adapted from the Cultural Formulation Interview (CFI)^74^, a validated framework designed to elicit culturally grounded understandings of health concerns and care-seeking. We selected the CFI because its emphasis on explanatory models (how individuals understand their health problems), cultural identity, and social context aligned with our aim of understanding how sociocultural factors shape healthcare barriers and facilitators. We collaboratively modified the protocol in two key ways: (1) broadening the framing to include both physical and mental health concerns, and (2) incorporating new domains focused on digital health technologies (e.g., app usage, telehealth, and perceptions of digital versus traditional care). These adaptations were developed through iterative team review. The resulting semi-structured guide maintained the CFI’s cultural emphasis while situating participants’ experiences within the contemporary digital healthcare landscape. The guide included branching logic so that if participants indicated no current health concerns, interviewers shifted focus to explore general healthcare system experiences, preventive care practices, and attitudes toward digital health.

The data-collection team comprised J.D., N.F., M.S., P.Z., Z.B., A.V., and J.L. English interviews were conducted by one interviewer (J.D.); Spanish interviews were co-led by JD and a bilingual interviewer (N.F.) to ensure accuracy. Interviewers were women. Coding was performed by a mixed-gender team with safeguards including double-coding and consensus reconciliation (see “Qualitative analysis”). Researcher roles, training, and demographic context are provided in the reflexivity statement (Supplemental Materials (SM), Supplementary Text 1). Participants were informed about study objectives; all interviews were audio- and video-recorded with consent, and interviewers wrote field notes after each session to support transcription accuracy and provide contextual data for analysis.

### Qualitative analysis

English interviews (*n* = 40) were transcribed verbatim by research staff. Spanish interviews (*n* = 7) were professionally translated and transcribed into English to ensure consistency across the dataset for coding purposes. Thematic analysis was conducted using MAXQDA 2022^75^, following both inductive and deductive approaches. Initial coding was essentially inductive, allowing salient patterns and themes to emerge directly from the data, while final theme development was guided by a deductive framework aligned with the study’s central research questions. To ensure analytic rigor, we implemented systematic coding procedures, team-based consensus building, and intercoder agreement assessment. The analytic process began with team members engaging in data familiarization, re-reading transcripts, and recording initial impressions. During the open coding phase, six team members, led by JD, independently coded a randomly selected subset of interviews, focusing on domains including healthcare barriers and facilitators, digital technology experiences, cultural and identity-related influences, and technology adoption patterns. Identity-related codes were also used to explore how social background shaped healthcare and DHT engagement (see Table 5 for the full code list).

**Table 5:** Overview of the theme and social identity code co-occurrence

	Social identity codes																	
Themes	BS	ND	I	AP	FE	WE	PB	F	LS	RS	MP	SO	FL	NO	GI	RE	A	SUM
Traditional healthcare barriers																		
Cultural	0	1	1	0	2	1	0	5	1	8	1	0	5	6	0	21	1	53
Financial	1	1	1	1	1	1	1	1	1	1	1	1	1	1	1	1	1	17
Structural	2	0	1	0	0	1	1	1	2	3	0	0	0	1	0	8	0	20
Bias/Discrim	0	0	0	0	0	0	0	0	0	0	0	0	0	0	0	0	0	0
Pandemic	0	0	0	0	2	0	0	1	0	1	1	0	0	0	0	0	0	5
Sum	3	1	5	0	4	7	2	8	4	12	3	0	5	8	0	31	1	94
Traditional healthcare facilitators																		
Relationships	1	0	0	0	3	4	0	7	0	1	1	0	0	3	0	2	2	24
Medical Proxy	0	0	3	0	2	3	0	1	0	1	0	0	2	0	0	0	0	12
Cultural Values	0	0	0	0	1	0	0	0	1	13	1	0	2	5	1	13	0	37
Non-DHT	0	0	0	0	0	0	0	0	0	0	0	0	0	0	0	0	0	0
Sum	1	0	3	0	6	7	0	8	1	15	2	0	4	8	1	15	2	73
DHT barriers																		
Relational Loss	0	0	0	0	1	0	0	0	0	0	0	0	0	0	0	0	0	1
eLiteracy/Access	0	0	0	0	0	0	0	0	0	0	0	0	0	0	0	0	0	0
Data security	0	0	1	0	0	0	0	0	0	1	0	0	0	0	0	0	0	2
Value/Cost	1	0	1	0	0	0	0	0	0	1	0	0	0	0	0	0	0	3
Sum	1	0	1	0	1	0	0	0	0	1	0	0	0	0	0	0	0	5
DHT facilitators																		
Access/Flex	0	0	0	0	0	2	0	0	0	0	0	0	0	0	0	0	0	2
Personalization	0	0	0	0	0	0	0	0	0	0	0	0	0	0	0	0	0	0
Engag/motivation	0	0	0	0	0	1	0	0	0	0	0	0	0	0	0	0	0	1
Financial	0	0	1	0	0	0	0	0	0	0	0	0	0	0	0	0	0	1
Identity Protect	0	0	0	0	0	0	0	0	0	0	0	0	0	0	0	0	0	0
Sum	0	0	1	0	0	2	0	0	0	0	0	0	0	0	0	0	0	4

Denotes the number of times specific social identity codes emerged with the specific themes. *Body* Body size, *ND* Neurodiversity, *I* Income, *AP* Appearance, *FE* Formal education, *WE* Work experience, *PB* Political belief, *F* Family, *LS* Legal status, *RS* Religious/spirituality, *MP* Mental/physical ability, *SO* Sexual orientation, *FL* First language, *NO* National Origin, *GO* Gender Identity, *RE* Race or Ethnicity, *A* Age, Sum indicates total codes in column/row.

Codes were iteratively reviewed and refined through team meetings, during which coders compared interpretations, consolidated codes, and developed preliminary themes. All interviews were double-coded to enhance trustworthiness, with discrepancies resolved via consensus. To assess intercoder reliability, we used MAXQDA’s document-level unit of analysis on 38 of 47 transcripts (81%). This approach tests whether coders applied the same codes within each interview regardless of textual location, which aligned with our focus on theme presence/absence at the participant level. Cohen’s *κ* values ranged from 0.72 to 0.92, indicating substantial to almost perfect agreement^76^. All discrepancies were resolved through consensus discussion, and reconciled datasets were used for theme development and quantitative integration. Complete details of the IRR procedures are provided in Supplementary Text 2.

### Quantitative data collection

To triangulate qualitative themes with a theory-guided profile of digital health readiness, we selected questionnaires that map to complementary layers of the digital divide—skills, use/familiarity, and trust/privacy—across traditional and digital care.

#### Health literacy

The Health Literacy Questionnaire (HLQ^47^; a 44-item self-report measure, was employed to assess health literacy across nine domains: feeling understood and supported by healthcare providers, having sufficient information to manage health, actively managing health, social support for health, appraisal of health information, ability to actively engage with healthcare providers, navigating the healthcare system, ability to find good health information, and understanding health information well enough to know what to do. Sample items include “I feel I have good information about health” and “I have enough information to help me deal with my health problems.” Items are scored on a 4-point scale (1 = *strongly disagree* to 4 = *strongly agree*) for the first five domains or a 5-point scale (1 = *cannot do or always difficult* to 5 = *always easy*) for the remaining four domains. Each domain is scored independently by summing the relevant items, with possible scores ranging from 4 to 16 for the first three domains, 5 to 20 for domains 4 and 5, and 5 to 25 for domains 6 to 9. Higher scores indicate greater health literacy in that specific area. The HLQ has demonstrated strong construct validity, reliability, and acceptability across diverse populations, with reliability estimates ranging from 0.77 to 0.90 and a well-supported nine-factor structure^47^. Consistent with HLQ design, no total score is computed; we retained all nine domains to preserve domain-specific variation that could be differentially shaped by social identities and context, which a composite would obscure^47,77^. In the present sample, Cronbach’s α ranged from 0.69 to 0.93 across the nine domains.

#### eHealth literacy

The eHealth Literacy Scale (eHEALS^78^; is an 8-item tool to assess individuals’ ability to find, understand, evaluate, and apply health information from electronic resources. Sample items include “I know how to find helpful health resources on the Internet” and “I can tell high-quality health resources from low-quality health resources on the Internet.” Items are scored on a 5-point Likert scale (1 = *strongly disagree* to 5 = *strongly agree*) with total scores ranging from 8 to 40. Higher scores indicate greater eHealth literacy. The eHEALS has demonstrated strong internal consistency across diverse populations, with Cronbach’s *α* ranging from 0.88 to 0.97^79^, and has been validated in multiple contexts and languages^80,81^. In the present sample, the Cronbach’s *α* was 0.91.

#### Technology use and comfort

The Technological Ease and Computer-based Habits Inventory (TECHI^82^; assessed general technology use, comfort, and familiarity. This 17-item measure was developed based on existing technology adoption literature and assesses participants’ general technological proficiency by measuring the frequency of use and comfort with various digital technologies. Sample items include “I use a smartphone on a daily basis” and “People close to me would refer to me as tech savvy.” Items are scored on a 6-point scale (0 = *strongly disagree* to 5 = *strongly agree*) with total scores ranging from 0 to 85. The TECHI yields a total score and two subscale scores (Extent of Use and Technological Competency and Patience). For this study, we used the total score to assess overall technological proficiency, which aligned with our research questions examining general comfort and capability with technology. Higher scores indicate greater technological proficiency. In the present sample, Cronbach’s α was 0.81 for the total score.

#### Privacy and security concerns

Finally, participants’ attitudes and concerns regarding mobile privacy and security were assessed using the Mobile Privacy and Security Concerns Scale^83^ a 3-item measure examining concerns about mobile health technology use. Items include “How concerned are you about privacy and security when sending or receiving messages on your mobile phone device?”, “How concerned are you about privacy and security from the apps you use on your mobile phone device?”, and “You limit the types of messages you send or receive on your mobile phone or device because you are worried about privacy and security.” All items used a 5-point Likert format. The first two items (messaging and apps) were coded 1 = *extremely concerned* to 5 = *not concerned at all*; the behavioral item (“I limit the types of messages…”) used 1 = *strongly disagree* to 5 = *strongly agree* and was reverse coded to align directionality. We computed a mean composite (possible range: 3–13) so that lower scores indicate greater privacy concern. The scale demonstrated good internal consistency in our sample (Cronbach’s *α* = 0.76).

### Quantitative analysis

Descriptive statistics were calculated for all quantitative variables. Given the modest sample size and potential non-normal/ordinal measurement, Spearman’s rank-order correlations (*r*_s_*)* were used to examine interrelationships among health literacy (HLQ domains), eHealth literacy (eHEALS), technological proficiency (TECHI), and privacy concerns. We report *r*_s_ with 95% CI estimated via the Bonett–Wright method, emphasizing magnitude and uncertainty rather than significance testing. Analyses are descriptive and hypothesis-generating rather than confirmatory. For links between theme presence (binary) and continuous measures, we likewise report *r*_s_ (a rank-based effect) with corresponding 95% CIs. See Supplementary Tables 2 and 3 for full correlation matrices, *p*-values, and CIs.

### Integration of qualitative and quantitative analysis

We employed a multi-method integration strategy to examine convergence and divergence between qualitative and quantitative findings. Each reconciled transcript was exported from MAXQDA as a binary code matrix indicating theme presence/absence for each participant. These were merged with quantitative survey data to create an integrated dataset. We used data triangulation to compare emergent themes with quantitative patterns and conducted Spearman’s correlations between participants’ theme endorsement patterns and survey scores (HLQ, eHEALS, TECHI). This enabled exploration of how individual differences in measured competencies related to specific barriers and facilitators described in narratives.

### Social identity intersections

To understand how intersecting identities shape healthcare experiences, we analyzed qualitative and quantitative data through an intersectionality lens. We used two complementary approaches. First (narrative salience, qualitative): we coded whether participants *spontaneously mentioned* a given social identity during the interview (e.g., race/ethnicity, income, education, first language, sexual orientation); this binary indicator (1 = mentioned at least once; 0 = not mentioned) indexes narrative salience. Using MAXQDA’s code-relations browser, we summarized co-occurrence of identity codes with barrier/facilitator themes to reflect how participants themselves made identity relevant. Second (measured identity–theme associations, quantitative): we correlated narrative salience with theme presence/absence at the participant level and with survey measures (HLQ, eHEALS, TECHI) using Spearman’s *r*_s_, with 95% CIs estimated via the Bonett–Wright method. For transparency, we complemented these with correlations between recorded demographics and theme presence. Recorded demographics were age, income, education, employment status, primary language, and U.S. birth status; to reduce sparsity while preserving ordinality, income was collapsed to three levels (Low ≤$50k; Middle $50–$100k; High ≥$100k) and education to three levels ( ≤ High school; Some college/Associate; Bachelor’s or higher). Given the sample composition, sex/gender and detailed race/ethnicity were summarized descriptively rather than modeled. Correlations used pairwise deletion; we report *r*_s_, 95% CI, two-tailed *p*, and Benjamini–Hochberg FDR-adjusted *q*. These analyses are exploratory and intended to aid interpretation rather than draw causal inferences.

## Supplementary information

Supplementary information

## Data Availability

The datasets generated and analyzed during the current study are not publicly available due to privacy and confidentiality restrictions for human participants, but are available from the corresponding author on reasonable request and with appropriate ethical approvals.

## Code availability

The custom code used for data analysis for the current study is available from the corresponding author on reasonable request upon reasonable request.
