# Supplementary material for: Caregiver perceptions of healthcare barriers across traditional and digital contexts: a mixed-methods analysis
Source: NPJ Digit Med. 2025 Dec 12;8:772. doi: 10.1038/s41746-025-02131-x (PMC12717138; doi:10.1038/s41746-025-02131-x)
Supplement: Supplementary file 1 — Supplementary information [file 41746_2025_2131_MOESM1_ESM.pdf]

Supplemental Materials

Supplementary Text 1. Reflexivity Statement

As a research team of six individuals, we acknowledge the importance of reflecting on our backgrounds, identities, and experiences that may influence our study on health disparities and digital tools. We come from diverse academic, cultural, and professional backgrounds, and our perspectives may shape our understanding of the subject matter. Within our diverse group, some have lived experiences related to healthcare disparities, while others have expertise in digital technology. These varied individual perspectives could impact our data collection, analysis, and interpretation.

We commit to ongoing self-reflection and open dialogue within our team to mitigate potential biases. We recognize that our beliefs and experiences should not influence the research process and findings. Additionally, we will document and discuss any personal biases or preconceptions that arise during the research to maintain the integrity of our analysis. We acknowledge our power and privilege as researchers and aim to use cultural proficiency to analyze participants' diverse experiences. We aim to conduct this research with transparency and rigor, acknowledging that our perspectives are part of the context of our study. By doing so, we seek to enhance the credibility and trustworthiness of our research findings.

Supplementary Text 2. Intercoder Reliability Procedures

Approach and Rationale

To assess intercoder reliability, we used MAXQDA's "document-level" unit of analysis on 38 of 47 transcripts (81%), with the remaining transcripts single-coded due to resource constraints. This partial IRR strategy reflects best practices in applied thematic analysis, balancing rigor and feasibility in large-scale qualitative research (Campbell et al., 2013; Nowell et al., 2017).

Document-Level vs. Segment-Level Analysis

Document-level analysis tests whether coders applied the same codes within the same interview, regardless of specific textual location. This criterion was chosen because our research questions and analytic approach focus on the presence or absence of themes at the participant level (e.g., whether a given barrier or facilitator emerged in a participant's account), rather than the precise sentence in which it was expressed.

In thematic analysis, coders often select different illustrative passages for the same underlying theme, which can yield artificially low segment-level reliability despite strong substantive agreement (Campbell et al., 2013; O'Connor & Joffe, 2020). We initially considered segment-level analysis but found it attenuated in our dataset, reflecting boundary differences in how coders selected illustrative passages for identical themes rather than substantive disagreement.

Reliability Statistics

At the document level, Cohen's κ values ranged from 0.72 to 0.92, indicating substantial to almost perfect agreement (Landis & Koch, 1977) that coders consistently identified the same themes across interviews.

Consensus and Integration Process

All coding discrepancies were resolved through consensus discussion prior to analysis. To integrate coders' work, second-coder files were merged using MAXQDA's Teamwork Import feature with the "use outer segment boundaries" option, preserving interpretive context and metadata. This process generated a single reconciled file per transcript that reflected all consensus codes, which formed the dataset used for subsequent thematic analysis and integration with quantitative variables.

Campbell, J. L., Quincy, C., Osserman, J., & Pedersen, O. K. (2013). *Coding in-depth semistructured interviews: Problems of unitization and intercoder reliability*. Sociological Methods & Research, 42(3), 294–320.

Nowell, L. S., Norris, J. M., White, D. E., & Moules, N. J. (2017). *Thematic analysis: Striving to meet the trustworthiness criteria*. International Journal of Qualitative Methods, 16(1), 1609406917733847.

O'Connor, C., & Joffe, H. (2020). *Intercoder reliability in qualitative research: Debates and practical guidelines*. International Journal of Qualitative Methods, 19, 1609406919899220.

Landis, J. R., & Koch, G. G. (1977). *The measurement of observer agreement for categorical data*. Biometrics, 33(1), 159–174.

Supplementary Table 1. Interrater Reliability Metrics for Double-Coded Transcripts

Coder 1	Coder 2	Document name	Agreements	Disagreements	Percent	Kappa (RK)
AV	JD	P3	129	40	76.33	0.76
AV	JD	P02	132	37	78.11	0.78
AV	JD	P1	141	28	83.43	0.83
PZ	AV	P4	131	38	77.51	0.78
PZ	AV	P5	138	31	81.66	0.82
ZB	PZ	P6	145	24	85.8	0.86
ZB	PZ	P8	134	35	79.29	0.79
ZB	JD	P9	127	42	75.15	0.75
ZB	JD	P10	137	32	81.07	0.81
AV	JD	P11	126	43	74.56	0.75
PZ	AV	P12	130	39	76.92	0.77
PZ	AV	P13	134	35	79.29	0.79
ZB	PZ	P14	131	38	77.51	0.78
ZB	PZ	P15	141	28	83.43	0.83
PZ	AV	P20	134	35	79.29	0.79
PZ	JD	P21	145	24	85.8	0.86
ZB	PZ	P22	141	28	83.43	0.83
ZB	PZ	P23	138	31	81.66	0.82
AV	JD	P27	146	23	86.39	0.86
AV	JD	P26	134	35	79.29	0.79
PZ	JD	P28	146	23	86.39	0.86
ZB	PZ	P30	155	14	91.72	0.92
ZB	PZ	P31	144	25	85.21	0.85
MS	JL	P34	126	43	74.56	0.75
MS	JL	P35	133	36	78.7	0.79
MS	JL	P36	145	24	85.8	0.86
MS	JL	P37	132	37	78.11	0.78
MS	JL	P38	146	23	86.39	0.86
MS	JL	P39	122	47	72.19	0.72
MS	JL	P40	137	32	81.07	0.81
MS	JL	P41	147	22	86.98	0.87
MS	JL	P42	144	25	85.21	0.85
MS	JL	P43	136	33	80.47	0.8
MS	JL	P44	132	37	78.11	0.78
MS	JL	P45	141	28	83.43	0.83
MS	JL	P46	145	24	85.8	0.86
MS	JL	P47	139	30	82.25	0.82
MS	JL	P48	139	30	82.25	0.82

Notes. This table presents interrater reliability (IRR) metrics for 38 of the 47 transcripts that were double coded as part of the thematic analysis. Each transcript was independently coded by two trained coders using a shared codebook. Agreements, disagreements, percent agreement, and Cohen's Kappa (κ) are reported. Kappa values ranged from 0.72 to 0.92, reflecting substantial to almost perfect agreement (Landis & Koch, 1977). The remaining 9 transcripts were also double-coded, but IRR could not be computed due to insufficient overlapping code segments or export issues; discrepancies for these were resolved through consensus.

Supplementary Table 2. *Spearman Rank-Order Correlations Between Quantitative Variables*

Variable	1	2	3	4	5	6	7	8	9	10	11	12
1. Feeling understood and supported by HCPs	—											
2. Having sufficient information to manage health	.62**	—										
3. Actively managing my health	.50**	.63**	—									
4. Social support for health	.58**	.54**	.81**	—								
5. Appraisal of health information	.41**	.35*	.54**	.58**	—							
6. Ability to actively engage with HCPs	.36*	.44**	.65**	.63**	.52**	—						
7. Navigating the healthcare system	.44**	.54**	.43**	.51**	.31*	.42**	—					
8. Ability to find good health information	.14	.34*	.36*	.49**	.46**	.60**	.53**	—				
9. Understanding health information	.09	.29*	.32*	.36*	.27	.58**	.29*	.64**	—			
10. TECHI	.28	.33	.19	.35**	.32*	.33*	-.02	.55***	.48***	—		
11. eHealth literacy	.32*	.45**	.57**	.62**	.66**	.64**	.38**	.60**	.33*	.45**	—	
12. Privacy/Security	-.16	-.15	-.08	-.08	.06	-.01	-.02	-.03	-.06	.02	.09	—

Note. HCPs = Health care providers. HLQ domains (1-9). $N = 47$. * = $p < .05$, ** = $p < .01$, *** = $p < .001$. See main manuscript for corresponding heatmap (Figure 4).

Supplementary Table 3. *Confidence Intervals for Spearman Rank-Order Correlations Between Quantitative Variables*

Variable	1	2	3	4	5	6	7	8	9	10	11	12
1. Feeling understood and supported by HCPs	—											
2. Having sufficient information to manage health	**[.38, .78]**	—										
3. Actively managing my health	**[.23, .70]**	**[.39, .78]**	—									
4. Social support for health	**[.33, .75]**	**[.28, .73]**	.**[.66, .90]**	—								
5. Appraisal of health information	**[.13, .63]**	**[.07, .59]**	**[.28, .73]**	.**[.33, .75]**	—							
6. Ability to actively engage with HCPs	**[.07, .59]**	**[.16, .66]**	**[.43, .80]**	**[.40, .79]**	**[.26, .71]**	—						
7. Navigating the healthcare system	**[.16, .66]**	**[.28, .72]**	**[.15, .64]**	**[.24, .70]**	**[.02, .55]**	**[.14, .64]**	—					
8. Ability to find good health information	[-.16, .41]	**[.05, .58]**	**[.07, .59]**	**[.21, .69]**	**[.19, .67]**	**[.36, .77]**	**[.27, .72]**	—				
9. Understanding health information	[-.21, .36]	**[.00, .54]**	**[.03, .56]**	**[.07, .59]**	[-.03, .52]	**[.33, .75]**	**[.00, .54]**	**[.40, .79]**	—			
10. TECHI	[-.02, .53]	[.04, .57]	[-.10, .46]	**[.14, .64]**	**[.03 .56]**	**[.04, .57]**	**[.04, .57]**	**[.29, .73]**	**[.21, .69]**	—		
11. eHealth literacy	**[.02, .56]**	**[.17, .66]**	**[.31, .74]**	**[.38, .78]**	**[.44, .81]**	**[.41, .80]**	**[.09, .61]**	**[.36, .77]**	**[.04, .57]**	**[.17, .66]**	—	
12. Privacy/Security	[-.43, .13]	[-.42, .15]	[-.36, .21]	[-.36, .21]	[-.23, .34]	[-.30, .28]	[-.31, .27]	[-.31, .26]	[-.35, .23]	[-.26, .32]	[-.21, .37]	—

Note. HCPs = Health care providers. HLQ domains (1-9). Values in brackets represent 95% confidence intervals. Confidence intervals calculated using Fisher's *r*-to-*z* transformation with standard error based on Bonett and Wright's formula. Bold values indicate significance. *N* = 47.

Supplementary Table 4. *Spearman Rank-Order Correlations Between Barrier/Facilitator Themes and Quantitative Variables*

Variable	1	2	3	4	5	6	7	8	9	10	11	12	13	14	15	16	17	18
**Traditional Healthcare Barriers**																		
1. Cultural belief barriers	—																	
2. Social barriers	.11	—																
3. System barriers	.08	.48**	—															
4. Provider discrimination	.22	.11	.06	—														
5. COVID-19 disruptions	.02	.21	.15	.17	—													
**Digital Health Barriers**																		
6. Relational connection loss	.11	.18	.08	.32*	.39**	—												
7. Digital literacy gaps	.11	.08	.11	-.04]	-.24	-.04	—											
8. Privacy concerns	.00	.08	.14	-.05	.05	-.02	.24	—										
9. Social-cultural inclusion	.15	-.02	-.18	-.12	-.17	.16	-.03	-.13	—									
**Traditional Health Facilitators**																		
10. Primary relationships	.08	.18	.08	.32*	.39**	-.02	-.04	-.02	.03	—								
11. Medical proximity	.11	.28	.02	.09	-.02	-.04	.05	-.14	.23	-.04	—							
12. Cultural factors	.11	-.14	-.23	-.02	.05	.35*	-.20	.07	.04	.35*	-.22	—						
13. Online connection	-.03	-.04	-.15	-.02	-.02	-.01	.17	.23	.10	-.01	-.12	.16	—					
**Digital Health Facilitators**																		
14. Access convenience	.13	.38**	.16	.08	.24	.33*	.17	.28	.09	.33*	.18	-.01	-.06	—				
15. Personalization	-.07	.15	.22	.26	.05	.06	.02	.08	-.01	.06	.15	-.01	.12	.24	—			
16. Engagement motivation	.01	.27	.22	-.30*	-.10	-.04	-.05	.30*	.23	-.04	.02	-.18	-.18	.42**	.13	—		
17. Cost-value assessment	.04	.13	.10	-.14	-.08	-.15	.03	.11	.25	-.15	.33*	-.22	-.11	.38**	.10	.52**	—	
18. Digital anonymity	.00	-.08	.09	.18	-.11	-.00	.34*	-.19	-.14	-.00	.13	-.11	-.02	-.01	.01	-.21	.20	—
**Health Literacy Variables**																		
Feeling understood by HCPs	-.10	-.10	.13	.21	-.14	.09	.04	.00	-.03	.09	.00	.13	-.12	.01	.28	-.02	.09	.25
Having sufficient info	-.17	-.05	.02	.19	-.08	.08	-.11	.09	-.09	.08	.09	-.02	-.13	.03	-.08	.00	.00	-.01
Actively managing health	-.21	-.05	.01	.05	-.03	-.07	.06	.14	-.02	-.07	.09	-.15	.07	.00	.19	.07	.11	-.05
Social support for health	-.06	-.21	.09	-.07	-.06	-.14	.12	.09	-.06	-.14	-.02	-.07	.10	-.03	.26	.08	.03	.01
Appraisal of information	-.08	-.07	.19	-.07	-.01	.03	-.12	-.06	-.18	.03	.08	.06	.13	-.02	.34*	.01	-.01	.13
Ability to engage with HCPs	-.02	-.06	.10	.13	.21	.23	.11	-.02	-.10	.23	.00	-.17	.07	.11	.15	-.03	-.01	.00
Navigating healthcare	.04	-.40**	-.15	.13	-.09	.00	-.05	.05	-.02	.00	.00	.14	-.07	-.02	.04	.09	-.03	-.13
Finding good information	.20	-.04	.17	-.06	.04	.08	.08	.04	-.14	.08	.03	-.22	-.07	.10	.11	.16	.07	-.07

Variable	1	2	3	4	5	6	7	8	9	10	11	12	13	14	15	16	17	18
Understanding information	.13	.09	.19	.07	.03	.14	.04	.06	.04	.14	.07	-.23	-.13	.30*	.14	.20	.17	-.11
TECHI	.19	-.10	.12	.15	-.17	.01	.06	.07	-.09	-.01	.02	-.09	-.13	.35	.21	.17	.21	.10
eHealth literacy	.07	-.10	.13	.19	.17	.20	-.14	.04	-.13	.20	-.02	.06	.20	.04	.22	-.05	-.03	-.08
Privacy/Security	.08	.06	-.03	.23	.19	.07	-.11	.07	-.09	.04	-.30	.02	.33*	.11	.04	-.03	-.16	.08

Note. HCPs = health care providers. $N = 47$. * = $p < .05$, ** = $\boldsymbol{p} < .01$, *** = $\boldsymbol{p} < .001$. See main manuscript for corresponding heatmap (Figure 5).

Supplementary Table 5. *95% Confidence Intervals Between Barrier/Facilitator Themes and Quantitative Variables*

Variable	1	2	3	4	5	6	7	8	9	10	11	12	13	14	15	16	17	18
**Traditional Healthcare Barriers**																		
1. Cultural belief barriers	—																	
2. Social barriers	[-.19, .38]	—																
3. System barriers	[-.21, .36]	**[.21, .69]**	—															
4. Provider discrimination	[-.08, .48]	[-.18, .39]	[-.23, .34]	—														
5. COVID-19 disruptions	[-.27, .31]	[-.08, .48]	[-.14, .42]	[-.13, .43]	—													
**Digital Health Barriers**																		
6. Relational connection loss	[-.18, .39]	[-.12, .45]	[-.21, .36]	**[.03, .56]**	**[.10, .61]**	—												
7. Digital literacy gaps	[-.18, .39]	[-.21, .36]	[-.18, .39]	[-.33, .25]	[-.50, .05]	[-.32, .25]	—											
8. Privacy concerns	[-.28, .29]	[-.21, .36]	[-.16, .41]	[-.33, .25]	[-.24, .33]	[-.30, .27]	[-.06, .50]	—										
9. Social-cultural inclusion	[-.15, .42]	[-.31, .27]	[-.45, .11]	[-.40, .17]	[-.44, .12]	[-.13, .43]	[-.31, .26]	[-.40, .16]	—									
**Traditional Health Facilitators**																		
10. Primary relationships	[-.22, .36]	[-.12, .45]	[-.21, .36]	**[.03, .56]**	**[.10, .61]**	[-.30, .27]	[-.32, .25]	[-.30, .27]	[-.26, .32]	—								
11. Medical proximity	[-.19, .38]	[-.02, .53]	[-.27, .31]	[-.21, .37]	[-.31, .27]	[-.32, .25]	[-.24, .34]	[-.41, .16]	[-.06, .49]	[-.32, .25]	—							
12. Cultural factors	[-.19, .38]	[-.41, .16]	[-.49, .06]	[-.30, .27]	[-.24, .33]	**[.06, .58]**	[-.46, .10]	[-.22, .35]	[-.25, .32]	**[.06, .59]**	[-.48, .08]	—						
13. Online connection	[-.32, .26]	[-.32, .25]	[-.42, .15]	[-.30, .27]	[-.30, .27]	[-.29, .27]	[-.12, .44]	[-.06, .49]	[-.19, .38]	[-.29, .28]	[-.40, .17]	[-.14, .43]	—					
**Digital Health Facilitators**																		
14. Access convenience	[-.17, .40]	**[.10, .61]**	[-.14, .43]	[-.21, .36]	[-.05, .50]	**[.04, .57]**	[-.12, .44]	[-.02, .53]	[-.20, .37]	**[.04, .57]**	[-.12, .44]	[-.29, .28]	[-.34, .24]	—				
15. Personalization	[-.35, .22]	[-.15, .42]	[-.07, .48]	[-.03, .51]	[-.24, .33]	[-.24, .34]	[-.27, .31]	[-.21, .36]	[-.30, .28]	[-.24, .34]	[-.15, .42]	[-.30, .28]	[-.18, .39]	[-.06, .49]	—			
16. Engagement motivation	[-.28, .29]	[-.02, .52]	[-.08, .48]	[-.54, -.00]	[-.38, .19]	[-.32, .25]	[-.33, .25]	**[.00, .54]**	[-.06, .49]	[-.32, .25]	[-.27, .31]	[-.44, .12]	[-.44, .12]	**[.13, .64]**	[-.16, .41]	—		
17. Cost-value assessment	[-.25, .33]	[-.17, .40]	[-.19, .37]	[-.41, .16]	[-.36, .21]	[-.42, .15]	[-.26, .32]	[-.18, .39]	[-.04, .51]	[-.42, .15]	**[.04, .57]**	[-.48, .07]	[-.38, .19]	**[.09, .61]**	[-.19, .38]	**[.26, .71]**	—	
18. Digital anonymity	[-.29, .29]	[-.36, .22]	[-.20, .37]	[-.12, .45]	[-.39, .18]	[-.29, .28]	**[.05, .58]**	[-.46, .11]	[-.42, .15]	[-.29, .28]	[-.17, .40]	[-.38, .19]	[-.31, .27]	[-.30, .28]	[-.28, .29]	[-.47, .09]	[-.10, .46]	—

Variable	1	2	3	4	5	6	7	8	9	10	11	12	13	14	15	16	17	18
**Health Literacy Variables**																		
Feeling understood by HCPs	[-.38, .19]	[-.38, .20]	[-.17, .40]	[-.09, .47]	[-.41, .16]	[-.21, .37]	[-.25, .33]	[-.28, .29]	[-.32, .26]	[-.21, .37]	[-.29, .29]	[-.16, .41]	[-.40, .17]	[-.28, .29]	[-.01, .53]	[-.30, .27]	[-.20, .37]	[-.05, .50]
Having sufficient information	[-.43, .13]	[-.34, .24]	[-.27, .30]	[-.11, .45]	[-.36, .21]	[-.22, .37]	[-.38, .19]	[-.21, .36]	[-.37, .20]	[-.22, .36]	[-.21, .37]	[-.31, .27]	[-.40, .17]	[-.26, .31]	[-.36, .22]	[-.28, .29]	[-.29, .29]	[-.30, .28]
Actively managing health	[-.47, .09]	[-.33, .24]	[-.28, .30]	[-.24, .33]	[-.32, .26]	[-.35, .22]	[-.23, .34]	[-.15, .41]	[-.31, .27]	[-.35, .22]	[-.21, .37]	[-.42, .15]	[-.22, .35]	[-.28, .29]	[-.11, .45]	[-.22, .35]	[-.18, .39]	[-.33, .24]
Social support for health	[-.34, .23]	[-.47, .09]	[-.20, .37]	[-.35, .22]	[-.34, .24]	[-.42, .15]	[-.17, .40]	[-.21, .37]	[-.34, .23]	[-.42, .15]	[-.30, .27]	[-.35, .22]	[-.19, .38]	[-.32, .26]	[-.03, .51]	[-.22, .36]	[-.26, .32]	[-.28, .30]
Appraisal of information	[-.36, .21]	[-.35, .22]	[-.11, .45]	[-.35, .22]	[-.29, .28]	[-.26, .31]	[-.40, .17]	[-.34, .23]	[-.45, .12]	[-.26, .31]	[-.21, .36]	[-.23, .34]	[-.17, .40]	[-.30, .27]	**[.05, .57]**	[-.28, .30]	[-.30, .28]	[-.17, .40]
Ability to engage with HCPs	[-.31, .27]	[-.34, .24]	[-.20, .37]	[-.17, .40]	[-.08, .48]	[-.07, .48]	[-.18, .39]	[-.30, .27]	[-.37, .20]	[-.07, .49]	[-.28, .29]	[-.44, .12]	[-.23, .35]	[-.18, .39]	[-.14, .42]	[-.32, .26]	[-.30, .28]	[-.29, .29]
Navigating healthcare system	[-.25, .32]	[-.62, -.11]	[-.42, .15]	[-.17, .40]	[-.36, .21]	[-.28, .29]	[-.33, .24]	[-.24, .33]	[-.31, .27]	[-.28, .29]	[-.29, .29]	[-.15, .42]	[-.35, .22]	[-.31, .27]	[-.25, .32]	[-.21, .37]	[-.32, .26]	[-.40, .16]
Finding good information	[-.09, .46]	[-.32, .25]	[-.12, .44]	[-.34, .24]	[-.25, .32]	[-.21, .36]	[-.21, .36]	[-.25, .33]	[-.42, .15]	[-.21, .36]	[-.26, .32]	[-.48, .08]	[-.35, .22]	[-.19, .38]	[-.19, .38]	[-.14, .43]	[-.23, .35]	[-.35, .22]
Understanding information	[-.17, .40]	[-.20, .37]	[-.10, .46]	[-.22, .35]	[-.26, .32]	[-.15, .42]	[-.25, .33]	[-.23, .34]	[-.25, .32]	[-.15, .42]	[-.23, .35]	[-.49, .07]	[-.40, .17]	**[.00, .54]**	[-.15, .41]	[-.09, .47]	[-.13, .44]	[-.39, .18]
TECHI	[-.11, .45]	[-.39, .19]	[-.18, .39]	[-.07, .48]	[-.44, .13]	[-.28, .30]	[-.23, .34]	[-.14, .43]	[-.52, .02]	[-.29, .28]	[-.27, .30]	[-.37, .20]	[-.40, .17]	**[.06 .58]**	**[-.08, .47]**	[-.13, .44]	[-.08, .47]	[-.19, .38]
eHealth literacy	[-.22, .35]	[-.37, .20]	[-.17, .40]	[-.11, .46]	[-.13, .44]	[-.10, .46]	[-.41, .16]	[-.25, .33]	[-.40, .17]	[-.10, .46]	[-.30, .27]	[-.23, .34]	[-.10, .46]	[-.25, .33]	[-.08, .48]	[-.33, .25]	[-.32, .26]	[-.36, .21]
Privacy/Security	[-.21, .36]	[-.23, .34]	[-.32, .26]	[-.25, .32]	[-.11, .45]	[-.23, .35]	[-.38, .19]	[-.23, .35]	[-.37, .20]	[-.25, .32]	[<.001,-.1]	[-.27, .30]	**[.04, .57]**	[-.18, .39]	[-.25, .33]	[-.31, .26]	[-.43, .13]	[-.21, .36]

Note. HCPs = health care providers. Values in brackets represent 95% confidence intervals. Confidence intervals calculated using Fisher's *r*-to-*z* transformation with standard error estimation based on Bonett and Wright's (2000) formula. Bold values indicate significance. *N* = 47.

Supplementary Table 6. *Spearman Rank-Order Correlations Between Barrier/Facilitator Themes and Social Identity Variables*

Variable	Body Size	Neuro-div.	Income	Appear.	Edu.	Work	Political	Family	Legal	Religion	Ability	Sex. Or.	First L.	Nat. Or.	Sex	Gender	Race/Eth.	Age
**Traditional Healthcare Barriers**																		
Cultural belief barriers	.29*	-.00	.41**	.24	.11	.38**	.16	-.04	.03	-.05	.03	.13	.26	.29*	.07	-.20	.41**	-.01
Social barriers	.23	.15	.22	-.03	-.02	-.01	-.07	.16	.12	-.10	-.22	-.15	-.03	.03	-.08	-.10	-.07	-.06
System barriers	.17	.15	.14	.12	-.04	.17	.07	-.06	.15	-.11	-.07	-.19	.09	.01	-.03	.07	.17	.02
Provider discrimination	.06	.01	-.02	.16	.04	.12	-.04	.22	.10	.04	.23	.18	-.11	.15	-.03	.01	.22	.24
COVID-19 disruptions	-.33*	.10	-.09	-.03	-.01	.04	.12	.27	.15	.09	-.22	-.13	.08	-.01	.35*	.27	.15	.14
**Digital Health Barriers**																		
Personal experience barriers	.11	-.11	.29*	-.04	.19	.25	.01	.26	-.01	-.10	.09	.06	-.03	.01	.43**	.07	.05	.05
Technology/professional access	-.05	.27	.20	.14	-.10	-.02	.10	-.05	.14	-.05	.11	-.18	-.26	-.13	.17	.00	-.04	.07
Personal attitudes/privacy	-.10	.27	.12	.05	.06	.21	-.06	-.08	-.07	.12	-.01	-.15	.06	-.06	.35*	.12	.10	.10
Social-cultural inclusion	.05	-.17	.20	-.04	.07	.11	-.01	.10	-.17	-.08	-.17	.12	.04	.09	-.17	-.20	.01	-.00
**Traditional Healthcare Facilitators**																		
Primary relationships	-.27	.07	-.33*	-.16	.03	.01	-.10	.13	-.15	.07	-.18	-.26	-.30*	-.23	.15	.15	.11	.00
Medical proximity	.19	-.05	.38**	-.14	.18	-.06	.08	.10	-.00	-.04	.01	-.08	.08	.25	-.07	-.15	-.11	-.09
Relational/cultural factors	-.03	-.06	-.15	-.19	.33*	.19	.07	.07	.02	.08	-.05	-.05	-.07	-.03	.09	-.01	.25	.00
Online connection	.05	.24	-.13	.09	.15	-.18	-.02	.13	-.03	.05	.18	.04	-.14	-.15	-.03	-.11	.02	-.10
**Digital Health Facilitators**																		
Access/convenience	.15	.27	.08	.04	.11	.25	-.13	.21	.01	.14	.00	.03	.03	-.11	.23	.09	.14	.09
Personalization	.23	.00	-.00	-.08	.20	.05	-.13	.27	-.05	.21	.19	.11	-.27	-.20	.13	.24	-.13	.16
Engagement/motivation	.07	.07	.11	-.04	.12	.22	-.09	-.02	-.09	.08	-.11	-.17	.07	-.11	.18	.02	-.06	.04
Cost/value assessment	.15	.03	.10	.21	.05	.12	-.01	.09	.18	.13	.05	-.12	.17	.02	.01	.17	-.02	.14
Digital anonymity	.14	.23	.07	.37**	.10	.04	.23	-.02	.21	-.24	.22	-.06	.02	-.05	-.08	.09	.17	.23

Note. **Narrative salience: explicit identity mentions co-occurring with themes.** Cells show the number of interviews in which a given identity category was explicitly mentioned in the same interview as the theme (document-level co-coding).Values in brackets represent 95% confidence intervals. Neuro-div. = neurodivergence; Appear. = appearance; Edu. = education; Sex. Or. = sexual orientation; First L. = first language; Nat. Or. = national origin; Race/Eth. = race/ethnicity. $N = 47$.$p < .05$. *$p < .01$. See main manuscript for corresponding heatmap (Figure 6).

Supplementary Table 7. *95% Confidence Intervals Between Barrier/Facilitator Themes and Social Identity Variables*

Variable	Body Size	Neuro-div.	Income	Appear.	Edu.	Work	Political	Family	Legal	Religion	Ability	Sex. Or.	First L.	Nat. Or.	Sex	Gender	Race/Eth.	Age
**Traditional Healthcare Barriers**																		
Cultural belief barriers	[-.01, .54]	[-.29, .28]	**[.13, .63]**	[-.05, .50]	[-.19, .38]	**[.09, .61]**	[-.14, .43]	[-.32, .25]	[-.26, .32]	[-.33, .24]	[-.26, .31]	[-.16, .41]	[-.04, .51]	[-.01, .54]	[-.22, .35]	[-.46, .10]	**[.13, .63]**	[-.30, .28]
Social barriers	[-.06, .49]	[-.15, .42]	[-.08, .48]	[-.32, .26]	[-.30, .27]	[-.30, .28]	[-.35, .22]	[-.14, .43]	[-.18, .39]	[-.38, .19]	[-.48, .08]	[-.42, .15]	[-.32, .26]	[-.26, .31]	[-.36, .21]	[-.38, .19]	[-.35, .22]	[-.34, .23]
System barriers	[-.13, .43]	[-.14, .42]	[-.16, .41]	[-.18, .39]	[-.32, .25]	[-.12, .44]	[-.22, .35]	[-.34, .23]	[-.15, .42]	[-.39, .19]	[-.35, .22]	[-.46, .11]	[-.21, .37]	[-.28, .30]	[-.32, .26]	[-.22, .35]	[-.13, .43]	[-.27, .31]
Provider discrimination	[-.23, .34]	[-.28, .30]	[-.30, .27]	[-.14, .43]	[-.25, .33]	[-.17, .40]	[-.32, .25]	[-.07, .48]	[-.20, .38]	[-.25, .32]	[-.07, .48]	[-.12, .44]	[-.39, .18]	[-.15, .42]	[-.31, .26]	[-.27, .30]	[-.08, .48]	[-.06, .49]
COVID-19 disruptions	[-.57, -.04]	[-.19, .38]	[-.37, .20]	[-.31, .26]	[-.30, .28]	[-.25, .32]	[-.18, .39]	[-.03, .52]	[-.15, .42]	[-.20, .37]	[-.48, .08]	[-.40, .17]	[-.21, .36]	[-.29, .28]	**[.06, .59]**	[-.02, .52]	[-.15, .42]	[-.16, .41]
**Digital Health Barriers**																		
Personal experience barriers	[-.19, .38]	[-.38, .19]	[-.00, .54]	[-.32, .25]	[-.11, .46]	[-.05, .51]	[-.28, .30]	[-.04, .51]	[-.29, .28]	[-.38, .19]	[-.20, .37]	[-.24, .34]	[-.32, .26]	[-.27, .30]	**[.15, .65]**	[-.22, .35]	[-.24, .33]	[-.24, .33]
Technology/professional access	[-.34, .24]	[-.02, .52]	[-.09, .46]	[-.16, .41]	[-.38, .19]	[-.30, .27]	[-.20, .38]	[-.33, .24]	[-.16, .41]	[-.33, .24]	[-.19, .38]	[-.45, .11]	[-.51, .04]	[-.40, .16]	[-.13, .43]	[-.29, .29]	[-.33, .25]	[-.23, .35]
Personal attitudes/privacy	[-.37, .20]	[-.02, .52]	[-.17, .40]	[-.24, .33]	[-.23, .34]	[-.09, .47]	[-.34, .23]	[-.36, .21]	[-.35, .22]	[-.18, .39]	[-.30, .28]	[-.42, .15]	[-.23, .34]	[-.34, .23]	**[.06, .59]**	[-.17, .40]	[-.20, .38]	[-.20, .38]
Social-cultural inclusion	[-.24, .34]	[-.44, .13]	[-.09, .47]	[-.32, .25]	[-.22, .35]	[-.19, .38]	[-.29, .28]	[-.19, .38]	[-.44, .13]	[-.36, .22]	[-.44, .13]	[-.18, .39]	[-.25, .32]	[-.21, .37]	[-.44, .13]	[-.46, .10]	[-.28, .30]	[-.29, .29]
**Traditional Healthcare Facilitators**																		
Primary relationships	[-.52, .03]	[-.22, .35]	[-.57, -.03]	[-.43, .13]	[-.26, .31]	[-.28, .29]	[-.38, .19]	[-.16, .40]	[-.42, .15]	[-.23, .35]	[-.45, .12]	[-.51, .04]	[-.54, -.01]	[-.49, .07]	[-.15, .42]	[-.14, .42]	[-.19, .38]	[-.29, .29]
Medical proximity	[-.10, .46]	[-.33, .24]	**[.09, .61]**	[-.42, .15]	[-.12, .44]	[-.34, .23]	[-.22, .36]	[-.19, .38]	[-.29, .29]	[-.32, .25]	[-.28, .29]	[-.36, .21]	[-.21, .36]	[-.04, .51]	[-.35, .22]	[-.42, .15]	[-.39, .18]	[-.37, .20]
Relational/cultural factors	[-.32, .26]	[-.34, .23]	[-.42, .15]	[-.45, .11]	**[.04, .57]**	[-.11, .45]	[-.23, .35]	[-.22, .35]	[-.27, .30]	[-.22, .36]	[-.33, .24]	[-.33, .24]	[-.35, .22]	[-.31, .26]	[-.20, .37]	[-.29, .28]	[-.04, .51]	[-.29, .29]
Online connection	[-.24, .33]	[-.06, .49]	[-.40, .17]	[-.21, .37]	[-.14, .42]	[-.44, .12]	[-.31, .27]	[-.16, .40]	[-.32, .26]	[-.24, .33]	[-.12, .44]	[-.25, .32]	[-.41, .15]	[-.42, .14]	[-.32, .26]	[-.38, .19]	[-.27, .30]	[-.38, .19]
**Digital Health Facilitators**																		
Access/convenience	[-.14, .42]	[-.03, .52]	[-.21, .36]	[-.25, .32]	[-.19, .38]	[-.05, .50]	[-.41, .16]	[-.09, .47]	[-.28, .30]	[-.15, .41]	[-.28, .29]	[-.26, .32]	[-.26, .32]	[-.39, .19]	[-.06, .49]	[-.20, .37]	[-.15, .41]	[-.21, .37]
Personalization	[-.06, .49]	[-.28, .29]	[-.29, .28]	[-.36, .21]	[-.10, .46]	[-.25, .33]	[-.40, .17]	[-.03, .52]	[-.33, .24]	[-.08, .48]	[-.10, .46]	[-.18, .39]	[-.52, .02]	[-.46, .10]	[-.16, .40]	[-.05, .50]	[-.40, .17]	[-.13, .43]
Engagement/motivation	[-.22, .35]	[-.23, .35]	[-.18, .39]	[-.32, .25]	[-.18, .39]	[-.08, .48]	[-.37, .20]	[-.31, .27]	[-.37, .20]	[-.21, .36]	[-.39, .18]	[-.44, .13]	[-.22, .35]	[-.38, .19]	[-.11, .45]	[-.27, .31]	[-.34, .23]	[-.25, .32]
Cost/value assessment	[-.15, .42]	[-.26, .31]	[-.20, .38]	[-.09, .47]	[-.24, .33]	[-.18, .39]	[-.30, .28]	[-.21, .37]	[-.12, .44]	[-.17, .40]	[-.24, .33]	[-.39, .18]	[-.13, .43]	[-.27, .31]	[-.28, .29]	[-.13, .43]	[-.30, .27]	[-.16, .41]
Digital anonymity	[-.15, .41]	[-.07, .48]	[-.23, .35]	**[.08, .60]**	[-.20, .37]	[-.25, .32]	[-.06, .49]	[-.30, .27]	[-.08, .47]	[-.50, .06]	[-.07, .48]	[-.34, .24]	[-.27, .31]	[-.34, .24]	[-.36, .21]	[-.21, .37]	[-.12, .44]	[-.07, .49]

Note. Narrative salience: explicit identity mentions co-occurring with themes. Cells show the number of interviews in which a given identity category was explicitly mentioned in the same interview as the theme (document-level co-coding).Values in brackets represent 95% confidence intervals. Neuro-div. = neurodivergence; Appear. = appearance; Edu. = education; Sex. Or. = sexual orientation; First L. = first language; Nat. Or. = national origin; Race/Eth. = race/ethnicity. Confidence intervals calculated using Fisher's *r*-to-*z* transformation with standard error estimation based on Bonett and Wright's (2000) formula. Bold values indicate significance. *N* = 47.

Supplementary Table 8. *Spearman Correlations Between Healthcare Themes and Demographic Variables*

	Traditional Healthcare Barriers					DHT Barriers			
**Demographic Variable**	**Cultural Beliefs**	**Financial Access**	**System Barriers**	**Provider Bias**	**COVID Impact**	**Relational Loss**	**Digital Gaps**	**Security Concerns**	**Value Assessment**
**Age**	-.03	-.19	.10	.08	-.34*	-.12	-.01	-.29*	-.03
**Employment Status**									
Full-time	.42**	-.15	-.07	-.12	-.11	-.11	.22	.03	.24
Part-time	-.07	-.04	-.16	-.17	-.14	.13	-.08	.05	.00
Student	-.03	-.00	.08	.05	.16	-.06	-.18	-.15	-.12
Homemaker	-.49***	.28	.19	-.02	.20	.10	-.12	.06	-.10
Unemployed	-.03	-.12	-.12	.18	-.11	-.07	-.09	.04	-.14
Retired	.06	.06	.19	.32*	.08	.02	.10	-.15	-.12
**Race/Ethnicity**									
White/Caucasian	-.18	-.13	-.10	-.13	-.18	.16	-.25	.04	.14
Black/African American	.15	.20	.11	.04	.21	-.20	.07	-.01	-.02
Asian	.23	-.15	-.07	-.01	-.01	.07	-.13	-.11	.24
American Indian/Alaska Native	.12	.24	.22	.21	-.01	.01	.27	-.11	-.08
Other Race	.06	-.03	.04	.12	-.01	-.23	.12	-.11	-.17
**Ethnicity**									
Hispanic	-.07	-.02	-.14	.12	.02	.14	-.27	-.19	.00
Non-Hispanic	.11	.13	.21	-.04	-.03	-.17	.28	.08	-.07
**Language**									
English	.08	.14	-.02	.32*	-.08	-.08	.09	.14	-.07
Spanish	-.09	.04	-.19	.21	.06	.22	-.31*	-.05	.00
Other Language	.40**	.01	-.14	.09	-.00	.21	.23	.04	.31*
**Nativity**									
Born in USA	.07	-.11	.03	-.17	-.09	-.07	.08	.31*	-.20
Not Born in USA	-.07	.11	-.03	.17	.09	.07	-.08	-.31*	.20
**Income Level**									
≤$50k	-.06	.01	.09	-.07	-.07	-.31*	-.06	-.12	-.17

Demographic Variable	Traditional Healthcare Barriers					DHT Barriers			
	**Cultural Beliefs**	**Financial Access**	**System Barriers**	**Provider Bias**	**COVID Impact**	**Relational Loss**	**Digital Gaps**	**Security Concerns**	**Value Assessment**
$50k-$100k	.06	-.13	-.16	.12	-.07	.06	.04	.08	.18
>$100k	.00	.20	.13	-.09	.25	.43**	.02	.08	-.00
**Education Level**									
High school or below	-.09	.12	.12	-.20	.27	-.15	-.02	-.15	-.19
Some college/AA	-.04	.15	.13	.05	.04	-.29*	.25	.28	-.26
Bachelor's or beyond	.09	-.22	-.20	.08	-.21	.37*	-.22	-.17	.36*

Table 8 continues...

Demographic Variable	Traditional Healthcare Facilitators				DHT Facilitators				
	**Support Networks**	**Knowledge Brokers**	**Cultural Resources**	**Digital Resources**	**Accessibility**	**Personalization**	**Engagement**	**Cost Value**	**Identity Protection**
**Age**	-.14	-.04	.06	-.06	-.32*	.12	-.15	.00	.35*
**Employment Status**									
Full-time	-.05	-.06	.16	-.05	.10	-.06	.08	.17	.05
Part-time	-.43**	.02	-.14	.04	-.26	-.20	.04	-.03	-.09
Student	.19	.10	.07	-.14	.03	.26	-.01	-.12	-.06
Homemaker	.23	.06	-.16	.07	.22	.00	.01	-.10	-.12
Unemployed	.08	-.06	.15	.02	-.23	.00	-.08	.03	-.07
Retired	.06	-.03	-.12	.04	-.00	.19	-.17	-.12	.39**
**Race/Ethnicity**									
White/Caucasian	-.26	.14	.15	.11	-.01	-.02	.08	.23	-.16
Black/African American	.32*	.00	-.05	-.23	.05	.12	.17	-.10	-.20
Asian	-.18	-.06	.10	.15	-.01	.02	-.12	-.08	-.04
American Indian/Alaska Native	.01	.14	-.01	.15	.12	.13	-.12	-.08	.58***
Other Race	.02	.06	-.12	-.08	-.03	-.08	-.24	-.02	.24
**Ethnicity**									

	Traditional Healthcare Facilitators				DHT Facilitators				
**Demographic Variable**	**Support Networks**	**Knowledge Brokers**	**Cultural Resources**	**Digital Resources**	**Accessibility**	**Personalization**	**Engagement**	**Cost Value**	**Identity Protection**
Hispanic	.02	.02	.13	-.03	.07	-.20	-.21	-.02	-.08
Non-Hispanic	-.04	.02	-.22	-.08	-.06	.24	.28	.07	.10
**Language**									
English	-.01	-.02	-.12	.26	.05	.32*	-.02	.21	.10
Spanish	-.02	.06	.07	-.15	.07	-.13	-.15	-.10	-.12
Other Language	.03	.04	.01	-.07	.25	.05	.26	.18	-.09
**Nativity**									
Born in USA	.00	-.18	.06	-.09	.10	-.02	.35*	.12	.16
Not Born in USA	.00	.18	-.06	.09	-.10	.02	-.35*	-.12	-.16
**Income Level**									
≤$50k	-.16	-.13	-.33*	-.20	-.15	-.06	.19	-.14	-.10
$50k-$100k	.14	.17	.26	.22	.06	.03	-.27	.25	.15
>$100k	.05	-.08	.14	-.04	.17	.05	.14	-.17	-.08
**Education Level**									
High school or below	.11	.19	-.16	-.07	-.06	-.03	-.11	-.05	-.09
Some college/AA	-.14	-.25	-.13	.07	.15	.08	.04	-.01	.22
Bachelor's or beyond	.06	.11	.21	-.01	-.10	-.06	.03	.04	-.15

Note. N = [sample size]. Correlations are Spearman's rho coefficients. *p < .05. **p < .01. ***p < .001. Theme abbreviations: Cultural Beliefs = Cultural Belief Barriers; Financial Access = Financial Access Constraints; System Barriers = Structural System Limitations; Provider Bias = Provider Bias and Discrimination; COVID Impact = Pandemic-Related Disruptions; Relational Loss = Relational Connection Loss; Digital Gaps = Digital Literacy and Access Gaps; Security Concerns = Data Security Concerns; Value Assessment = Value-Cost Assessment; Support Networks = Supportive Relationship Networks; Knowledge Brokers = Healthcare Knowledge Brokers; Cultural Resources = Cultural Resource Integration; Digital Resources = Non-Clinical Digital Resources; Accessibility = Accessibility and Flexibility; Personalization = Personalized Health Management; Engagement = Engagement Mechanisms; Cost Value = Financial Incentivization; Identity Protection = Identity Protection.

Supplementary Table 9. *95% Confidence Intervals for Spearman Correlations Between Healthcare Themes and Demographic Characteristics*

	Traditional Healthcare Barriers					DHT Barriers			
**Demographic Variable**	**Cultural Beliefs**	**Financial Access**	**System Barriers**	**Provider Bias**	**COVID Impact**	**Relational Loss**	**Digital Gaps**	**Security Concerns**	**Value Assessment**
**Age**	[-.32, .26]	[-.46, .10]	[-.20, .37]	[-.21, .36]	[-.58, -.05]	[-.39, .18]	[-.29, .28]	[-.54, .00]	[-.30, .27]
**Employment Status**									
Full-time	**[.14, .64]**	[-.42, .14]	[-.35, .22]	[-.39, .18]	[-.38, .19]	[-.38, .19]	[-.08, .48]	[-.26, .32]	[-.05, .50]
Part-time	[-.35, .22]	[-.32, .25]	[-.46, .10]	[-.43, .13]	[-.41, .16]	[-.16, .41]	[-.36, .21]	[-.24, .33]	[-.29, .29]
Student	[-.31, .26]	[-.29, .28]	[-.21, .36]	[-.24, .33]	[-.14, .43]	[-.34, .23]	[-.45, .11]	[-.42, .14]	[-.39, .18]
Homemaker	[-.69, -.22]	[-.02, .53]	[-.11, .45]	[-.31, .27]	[-.09, .46]	[-.20, .37]	[-.40, .17]	[-.24, .34]	[-.38, .20]
Unemployed	[-.32, .26]	[-.39, .18]	[-.39, .18]	[-.12, .44]	[-.39, .18]	[-.35, .22]	[-.37, .20]	[-.25, .33]	[-.42, .15]
Retired	[-.24, .34]	[-.23, .35]	[-.11, .45]	**[.03, .56]**	[-.22, .36]	[-.27, .30]	[-.19, .38]	[-.42, .14]	[-.39, .18]
**Race/Ethnicity**									
White/Caucasian	[-.44, .12]	[-.40, .17]	[-.37, .20]	[-.40, .16]	[-.44, .12]	[-.14, .43]	[-.51, .04]	[-.25, .32]	[-.15, .42]
Black/African American	[-.14, .42]	[-.10, .46]	[-.18, .39]	[-.25, .32]	[-.08, .47]	[-.46, .10]	[-.22, .35]	[-.29, .28]	[-.31, .27]
Asian	[-.07, .49]	[-.42, .14]	[-.35, .23]	[-.29, .28]	[-.29, .28]	[-.23, .35]	[-.40, .17]	[-.38, .19]	[-.05, .50]
American Indian/Alaska Native	[-.17, .40]	[-.05, .50]	[-.07, .48]	[-.09, .47]	[-.29, .28]	[-.28, .30]	[-.03, .52]	[-.38, .19]	[-.36, .21]
Other Race	[-.24, .34]	[-.32, .26]	[-.25, .32]	[-.18, .39]	[-.29, .28]	[-.48, .07]	[-.18, .39]	[-.38, .19]	[-.44, .13]
**Ethnicity**									
Hispanic	[-.35, .22]	[-.31, .27]	[-.41, .16]	[-.17, .40]	[-.27, .31]	[-.15, .41]	[-.52, .02]	[-.46, .10]	[-.29, .29]
Non-Hispanic	[-.18, .39]	[-.16, .40]	[-.08, .47]	[-.33, .25]	[-.31, .26]	[-.44, .12]	[-.02, .53]	[-.21, .36]	[-.35, .22]
**Language**									
English	[-.21, .36]	[-.16, .41]	[-.31, .27]	**[.03, .56]**	[-.36, .22]	[-.36, .22]	[-.21, .37]	[-.16, .41]	[-.35, .22]
Spanish	[-.37, .20]	[-.25, .32]	[-.45, .11]	[-.09, .47]	[-.23, .34]	[-.07, .48]	[-.55, -.02]	[-.33, .25]	[-.29, .29]
Other Language	**[.12, .62]**	[-.28, .30]	[-.41, .15]	[-.21, .36]	[-.29, .29]	[-.09, .47]	[-.07, .49]	[-.25, .33]	**[.02, .55]**
**Nativity**									
Born in USA	[-.22, .35]	[-.39, .18]	[-.26, .31]	[-.44, .13]	[-.36, .21]	[-.35, .23]	[-.21, .36]	**[.02, .55]**	[-.47, .09]
Not Born in USA	[-.35, .22]	[-.18, .39]	[-.31, .26]	[-.13, .44]	[-.21, .36]	[-.23, .35]	[-.36, .21]	[-.55, -.02]	[-.09, .47]
**Income Level**									

	Traditional Healthcare Barriers					DHT Barriers			
**Demographic Variable**	**Cultural Beliefs**	**Financial Access**	**System Barriers**	**Provider Bias**	**COVID Impact**	**Relational Loss**	**Digital Gaps**	**Security Concerns**	**Value Assessment**
≤$50k	[-.34, .24]	[-.28, .30]	[-.21, .37]	[-.35, .23]	[-.36, .22]	[-.55, -.01]	[-.34, .24]	[-.40, .18]	[-.44, .13]
$50k-$100k	[-.24, .34]	[-.40, .17]	[-.43, .14]	[-.18, .40]	[-.36, .22]	[-.23, .35]	[-.25, .33]	[-.22, .36]	[-.12, .45]
>$100k	[-.29, .29]	[-.10, .46]	[-.17, .40]	[-.38, .20]	[-.05, .51]	**[.15, .65]**	[-.27, .31]	[-.22, .36]	[-.29, .29]
**Education Level**									
High school or below	[-.37, .21]	[-.17, .40]	[-.17, .40]	[-.46, .10]	[-.02, .52]	[-.42, .15]	[-.30, .27]	[-.42, .15]	[-.45, .11]
Some college/AA	[-.32, .25]	[-.14, .42]	[-.17, .40]	[-.24, .33]	[-.25, .32]	[-.54, .00]	[-.05, .50]	[-.01, .53]	[-.51, .04]
Bachelor's or beyond	[-.20, .37]	[-.48, .08]	[-.46, .10]	[-.22, .36]	[-.47, .09]	**[.08, .60]**	[-.48, .08]	[-.44, .12]	**[.07, .59]**

Table 9 continues...

	Traditional Healthcare Facilitators				DHT Facilitators				
**Demographic Variable**	**Support Networks**	**Knowledge Brokers**	**Cultural Resources**	**Digital Resources**	**Accessibility**	**Personalization**	**Engagement**	**Cost Value**	**Identity Protection**
**Age**	[-.41, .16]	[-.32, .25]	[-.24, .34]	[-.34, .23]	[-.56, -.03]	[-.18, .39]	[-.42, .14]	[-.29, .29]	**[.06, .59]**
**Employment Status**									
Full-time	[-.34, .24]	[-.34, .24]	[-.14, .43]	[-.33, .24]	[-.20, .37]	[-.35, .23]	[-.21, .36]	[-.12, .44]	[-.25, .33]
Part-time	[-.64, -.15]	[-.27, .30]	[-.41, .16]	[-.25, .33]	[-.51, .03]	[-.46, .10]	[-.26, .32]	[-.32, .26]	[-.37, .20]
Student	[-.11, .45]	[-.20, .37]	[-.23, .35]	[-.41, .15]	[-.26, .31]	[-.03, .51]	[-.30, .28]	[-.39, .18]	[-.34, .24]
Homemaker	[-.06, .49]	[-.23, .35]	[-.43, .14]	[-.22, .35]	[-.07, .48]	[-.29, .29]	[-.28, .30]	[-.38, .20]	[-.39, .18]
Unemployed	[-.21, .36]	[-.34, .23]	[-.15, .42]	[-.27, .31]	[-.48, .07]	[-.29, .29]	[-.36, .22]	[-.26, .32]	[-.35, .22]
Retired	[-.23, .34]	[-.32, .26]	[-.40, .17]	[-.25, .32]	[-.29, .28]	[-.11, .45]	[-.44, .13]	[-.39, .18]	**[.10, .61]**
**Race/Ethnicity**									
White/Caucasian	[-.51, .04]	[-.16, .41]	[-.15, .42]	[-.19, .38]	[-.29, .28]	[-.30, .27]	[-.22, .36]	[-.07, .49]	[-.43, .14]
Black/African American	**[.03, .56]**	[-.29, .29]	[-.34, .24]	[-.49, .07]	[-.24, .33]	[-.18, .39]	[-.12, .44]	[-.38, .19]	[-.46, .10]
Asian	[-.45, .12]	[-.34, .24]	[-.20, .37]	[-.14, .42]	[-.30, .28]	[-.27, .31]	[-.39, .18]	[-.36, .21]	[-.32, .25]
American Indian/Alaska Native	[-.28, .30]	[-.16, .41]	[-.29, .28]	[-.14, .42]	[-.18, .39]	[-.16, .40]	[-.39, .18]	[-.36, .21]	**[.33, .75]**
Other Race	[-.27, .31]	[-.23, .34]	[-.40, .17]	[-.36, .22]	[-.31, .26]	[-.36, .21]	[-.50, .05]	[-.30, .27]	[-.06, .50]
**Ethnicity**									

	Traditional Healthcare Facilitators				DHT Facilitators				
**Demographic Variable**	**Support Networks**	**Knowledge Brokers**	**Cultural Resources**	**Digital Resources**	**Accessibility**	**Personalization**	**Engagement**	**Cost Value**	**Identity Protection**
Hispanic	[-.27, .31]	[-.27, .31]	[-.16, .40]	[-.32, .26]	[-.23, .35]	[-.46, .09]	[-.47, .09]	[-.31, .27]	[-.36, .21]
Non-Hispanic	[-.33, .25]	[-.27, .31]	[-.48, .08]	[-.36, .21]	[-.35, .23]	[-.06, .50]	[-.02, .53]	[-.22, .35]	[-.19, .38]
**Language**									
English	[-.30, .28]	[-.30, .27]	[-.40, .17]	[-.04, .51]	[-.25, .33]	**[.03, .56]**	[-.30, .27]	[-.09, .47]	[-.19, .38]
Spanish	[-.30, .27]	[-.23, .34]	[-.22, .35]	[-.42, .15]	[-.22, .35]	[-.41, .16]	[-.42, .14]	[-.37, .20]	[-.40, .17]
Other Language	[-.26, .31]	[-.25, .33]	[-.28, .29]	[-.35, .22]	[-.05, .50]	[-.24, .33]	[-.04, .51]	[-.12, .44]	[-.37, .20]
**Nativity**									
Born in USA	[-.29, .29]	[-.45, .11]	[-.24, .34]	[-.37, .20]	[-.20, .37]	[-.30, .27]	**[.06, .58]**	[-.18, .39]	[-.13, .43]
Not Born in USA	[-.29, .29]	[-.11, .45]	[-.34, .24]	[-.20, .37]	[-.37, .20]	[-.27, .30]	[-.58, -.06]	[-.39, .18]	[-.43, .13]
**Income Level**									
≤$50k	[-.43, .14]	[-.40, .17]	[-.58, -.04]	[-.46, .10]	[-.43, .15]	[-.34, .24]	[-.11, .46]	[-.42, .16]	[-.38, .20]
$50k-$100k	[-.16, .41]	[-.13, .44]	[-.04, .52]	[-.08, .48]	[-.24, .34]	[-.26, .32]	[-.52, .02]	[-.05, .50]	[-.15, .42]
>$100k	[-.24, .34]	[-.36, .22]	[-.16, .41]	[-.32, .26]	[-.12, .44]	[-.25, .33]	[-.16, .41]	[-.44, .13]	[-.36, .21]
**Education Level**									
High school or below	[-.18, .39]	[-.11, .45]	[-.43, .14]	[-.35, .22]	[-.34, .23]	[-.31, .26]	[-.39, .18]	[-.33, .24]	[-.37, .20]
Some college/AA	[-.41, .16]	[-.50, .05]	[-.40, .17]	[-.23, .35]	[-.15, .42]	[-.21, .36]	[-.26, .32]	[-.30, .28]	[-.07, .48]
Bachelor's or beyond	[-.24, .34]	[-.18, .39]	[-.08, .48]	[-.30, .28]	[-.38, .19]	[-.34, .24]	[-.26, .32]	[-.25, .33]	[-.42, .15]

Note. N = [sample size]. Confidence intervals are for Spearman's rho coefficients using Fisher's r-to-z transformation. Intervals containing zero indicate non-significant correlations. Intervals excluding zero indicate significant correlations. Theme abbreviations: Cultural Beliefs = Cultural Belief Barriers; Financial Access = Financial Access Constraints; System Barriers = Structural System Limitations; Provider Bias = Provider Bias and Discrimination; COVID Impact = Pandemic-Related Disruptions; Relational Loss = Relational Connection Loss; Digital Gaps = Digital Literacy and Access Gaps; Security Concerns = Data Security Concerns; Value Assessment = Value-Cost Assessment; Support Networks = Supportive Relationship Networks; Knowledge Brokers = Healthcare Knowledge Brokers; Cultural Resources = Cultural Resource Integration; Digital Resources = Non-Clinical Digital Resources; Accessibility = Accessibility and Flexibility; Personalization = Personalized Health Management; Engagement = Engagement Mechanisms; Cost Value = Financial Incentivization; Identity Protection = Identity Protection.
